# In vivo high-speed microscopy of microbubbles in the chorioallantoic membrane model

**DOI:** 10.7150/thno.91232

**Published:** 2024-02-17

**Authors:** Rojin Anbarafshan, Carly Pellow, Kevin Kiezun, Hon Leong, David E. Goertz

**Affiliations:** 1 Department of Medical Biophysics, University of Toronto, Toronto, M5G 1L7, Canada.; 2 Sunnybrook Research Institute, Toronto, M4N 3M5, Canada.

**Keywords:** cavitation, high-speed imaging, intravital microscopy, in vivo, microbubbles, therapeutic ultrasound, vascular bioeffects

## Abstract

**Rationale:** The acoustic stimulation of microbubbles within microvessels can elicit a spectrum of therapeutically relevant bioeffects from permeabilization to perfusion shutdown. These bioeffects ultimately arise from complex interactions between microbubbles and microvascular walls, though such interactions are poorly understood particularly at high pressure, due to a paucity of direct *in vivo* observations. The continued development of focused ultrasound methods hinges in large part on establishing links between microbubble-microvessel interactions, cavitation signals, and bioeffects.

**Methods:** Here, a system was developed to enable simultaneous high-speed intravital imaging and cavitation monitoring of microbubbles *in vivo* in a chorioallantoic membrane model. Exposures were conducted using the clinical agent Definity^TM^ under conditions previously associated with microvascular damage (1 MHz, 0.5-3.5 MPa, 5 ms pulse length).

**Results:** Ultrasound-activated microbubbles could be observed and were found to induce localized wall deformations that were more pronounced in smaller microvessels and increased with pressure. A central finding was that microbubbles could extravasate from microvessels (from 34% of vessels at 1 MPa to 79% at 3 MPa) during insonation (94% within 0.5 ms) and that this occurred more frequently and in progressively larger microvessels (up to 180 µm) as pressure was increased. Following microbubble extravasation, transient or sustained red blood cell leakage ensued at the extravasation site in 96% of cases for pressures ≥1 MPa.

**Conclusions:** The results here represent the first high-speed *in vivo* investigation of high-pressure focused ultrasound-induced microbubble-microvessel interactions. This data provides direct evidence that the process of activated microbubble extravasation can occur *in vivo* and that it is linked to producing microvessel wall perforations of sufficient size to permit red blood cell leakage. The association of red blood cell leakage with microbubble extravasation provides mechanistic insight into the process of microvessel rupture, which has been widely observed in histology.

## Introduction

Focused ultrasound (FUS) in combination with circulating intravascular microbubbles is rapidly emerging as a non-invasive method for inducing a range of therapeutically relevant bioeffects 1, 2. These effects are highly dependent on ultrasound exposure conditions, and in particular, the microbubble-vessel interactions that ensue 3. The behavior of microbubbles within microvessels is complex, and linking the emitted acoustic signatures with bioeffects could potentially provide a means by which to monitor and control treatments 4.

The most developed application for microbubble-mediated FUS is drug delivery, where typical ultrasound exposures consist of millisecond scale pulses with pressures of 100s of kPa 5. In this exposure regime, microbubbles undergo predominantly stable oscillations (cavitation) that induce microvascular wall deformations and microstreaming 6 which can lead to transient increases in microvascular permeability and facilitate the drug transport into the extravascular space 7-9. As FUS pressures are increased, microbubble oscillations become more pronounced, and inertial behaviors such as jetting and violent collapse can occur 10-12. These can elicit a broader spectrum of bioeffects which range from inflammation 13, 14 to microvascular wall damage that can be accompanied by platelet activation 15, 16 and red blood cell (RBC) extravasation or leakage 5, 17. The latter effect has been attributed to a localized degradation of the integrity of the microvascular walls, often referred to as rupture. Though the details of the RBC extravasation process remain to be elucidated, there is considerable evidence that it is associated with violent inertial cavitation, which produces broad bandwidth acoustic bubble emissions 15. For drug delivery applications where the objective is to preserve the targeted tissue, these bioeffects are generally considered undesirable and are avoided by implementing bubble emission control algorithms that minimize inertial cavitation and maximize other features (such as sub- and ultra-harmonics) 18-21.

At higher pressures (typically above 1 MPa), irreversible blood flow shutdown can be induced. The mechanisms responsible for this remain to be established, and while it has been linked in some studies with thrombosis 11, 16 and endothelial cell damage 22, 23, the processes involved may depend on the precise exposure conditions employed, which have varied considerably in the literature. Specifically, antivascular ultrasound exposure conditions have employed a range of frequencies (0.24-5 MHz), peak negative pressures (0.18-10 MPa), modes of continuous wave and pulse waves with varying burst durations (0.0015-100 ms), and pulse repetition frequencies (0.01-3 kHz) 24. If a sufficiently pronounced (i.e. persistent) microvascular shutdown occurs within a region of tissue, ischemic necrosis will ensue, which is the basis of an emerging therapeutic approach referred to as nonthermal ablation or antivascular ultrasound 25-29. In the setting of oncology, preclinical work has demonstrated that antivascular therapy can produce antitumor effects on its own, as well as profoundly enhance the effects of radiotherapy 22, 23, 30-33, chemotherapy 34-37, antiangiogenic therapy 38-40, and immunotherapy 41.

Thus, at higher pressures, microbubble-microvessel interactions can produce bioeffects that are deleterious in the setting of drug delivery but have the potential to be harnessed in antivascular therapy. Despite their significance, there is at present only a limited understanding of microbubble-microvessel interactions and their relation to induced bioeffects at pressures approaching or above 1 MPa 16, 42-44. With respect to microvascular rupture for example, this has been inferred primarily by histology 37, 45 or microscopy 37, 46-50 based on the presence of perivascular RBCs. Important insights into microbubble-microvessel interactions have been made using high-speed microscopy observations of bubbles insonated with short (2-3 cycles) pulses within transparent *ex vivo* microvascular bed preparations. In a rat cecum model using a 1 kframe/s stroboscopic microscopy approach, it was demonstrated that stimulated intravascular bubbles could induce microvascular wall deformations and undergo jetting directed at the vessel walls 50. These observations were extended in studies of rat mesentery using 3 Mframe/s microscopy, thereby further highlighting the roles of jetting and induced microvascular wall invaginations 51, 52. Histology and electron microscopy indicated endothelial cells could be damaged and detached from the underlying basement membrane, although microvessel rupture was not detected 50-53. A subsequent analysis of bubble dynamics indicated that the wall deformations were indicative of high levels of circumferential strain, particularly during invaginations. While valuable insights were obtained in these studies, a notable limitation was that *ex vivo* configurations were employed with vessels that were devoid of RBCs. Although intravascular dye leakage was observed, the connection to vessel rupture to the degree that RBC extravasation occurred could not be determined. It is also notable that there is evidence that the proximity of RBCs can modify bubble behavior 54, 55, with simulations indicating that the pressures exerted by bubble oscillations upon microvessel walls are impacted by the presence of RBCs 56. Therefore, the nature of bubble-wall interactions that induce microvascular rupture on a scale sufficient to result in RBC extravasation remains to be elucidated. It is of particular interest to understand this process in the setting of millisecond scale pulses that are typically employed in FUS microbubble therapy, as recent work in compliant microchannels has shown that bubble behavior can be complex and evolve during the course of longer pulses 57.

**Figure 1:**
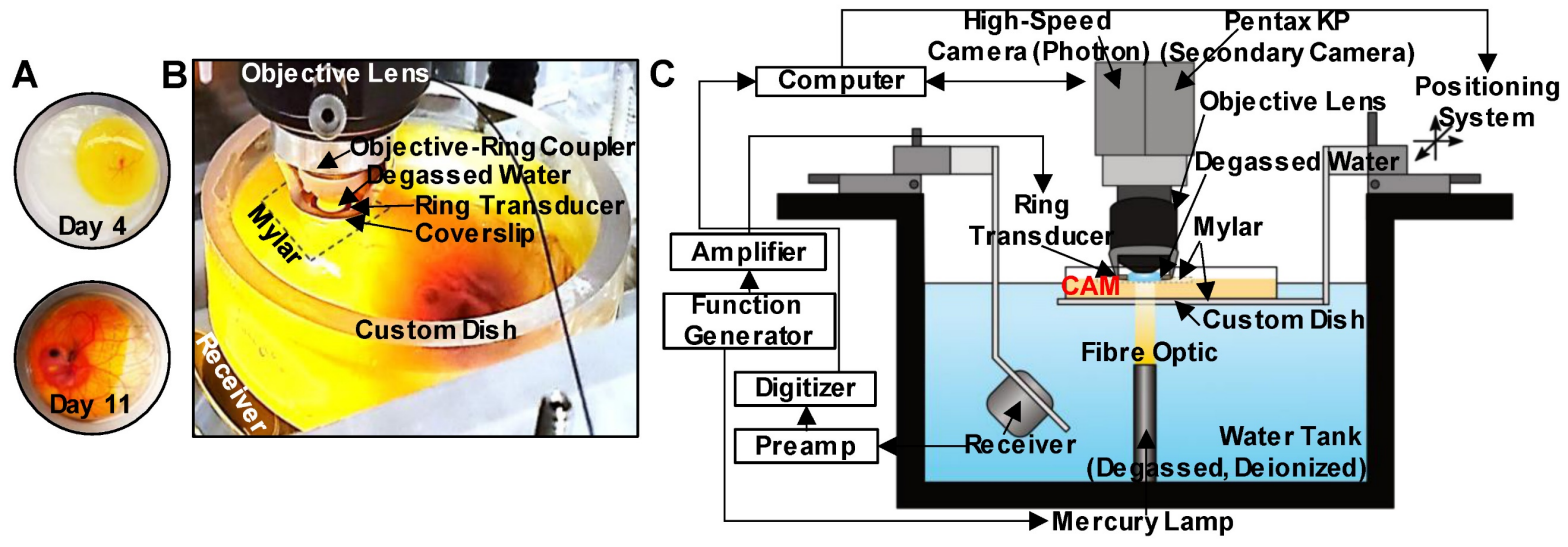
** Experimental configuration. (A)** The custom-made dish for ex ovo embryo cultivation with a mylar bottom and open top, displaying CAM appearance on days 4 and 11. **(B)** Photograph of the key components of the experimental apparatus. The bottom surface of the custom dish is made from mylar for optical (enabling the passage of light from the mercury lamp situated below the dish) and acoustic transparency (for cavitation detection with the co-aligned receiver below the dish). An additional mylar sheet is placed over the top of the CAM to stabilize the sample, as well as to keep the ring transducer and objective lens outside of the sample, and the objective lens with the objective-ring coupler is lowered over the top of the mylar such that the ring transducer rests on the mylar. A droplet of degassed water gets placed inside the ring transducer and couples with the water immersion objective lens for imaging. The foci of the ring transducer and receiver are co-aligned and are also aligned with the imaging focal plane of the objective lens. **(C)** Schematic of the full experimental configuration.

In this study, we investigate the interactions of microbubbles and microvessels arising from higher pressure millisecond scale ultrasound pulse stimulation in an *in vivo* duck embryo chorioallantoic membrane (CAM) *ex ovo* model. A high-speed intravital microscopy apparatus was developed to properly position the CAM tissue while exposing it to ultrasound and monitoring acoustic emissions (***Figure 1***). Circulating Definity^TM^ microbubbles were stimulated by ultrasound (PZT-4 ring transducer; 10 mm outer diameter, 8.5 mm inner diameter, 1.1 mm thickness, ~1 mm focal depth, 1 MHz center frequency) with simultaneous high-speed intravital imaging on either a single vessel (10k fps, 2 µs shutter speed) or vascular network (3k fps, 1 µs shutter speed) scale. A second camera (60 fps) was employed to examine longer timescales (on the order of minutes) to provide insight into sustained effects. Finally, passive cavitation detection (broadband receiver; 1” diameter, 1.63” focal length, 1 MHz center frequency) was employed to gain insight into the cavitation signatures associated with optical events.

## Materials and methods

### Animal model preparation

All animal procedures were approved and conducted in compliance with the Animal Care Committee guidelines at Sunnybrook Research Institute, Canada. Fertilized duck eggs (Khaki Campbell and White Layer Hybrid ducks; Weber Farm, Ontario, Canada) were placed in a rotating incubator for 4 days (Maru Deluxe, Rcom, Wichita, KS, USA) at 38°C and 45% humidity. On day 4, embryos were removed from their shells according to a standard *ex ovo* culturing method 58 and placed into custom-made dishes. These custom dishes were fabricated to offer a sterile environment for a living embryo with an optically and acoustically transparent window to accommodate simultaneous high-speed intravital imaging and cavitation detection of microbubbles in CAM vessels. The dishes were made from 10 cm diameter polycarbonate rings (McMaster-Carr, Aurora, OH, USA), with the bottom surface approximately 20 mm away from the transducer and covered with 76 µm thickness mylar film (Polyethylene terephthalate, McMaster-Carr, Aurora, OH, USA) to allow for the passage of both light and sound (notably to reduce the presence of acoustic reflections and the formation of standing waves). Embryos cultivated in the custom dishes were then placed in another incubator (model 7002-25, heated incubator, Caron, Marietta, OH, USA) at 37ºC and 30% humidity for 7-9 days, until the CAM had grown sufficiently over the albumin to provide an optically transparent region of interest. Experiments were then performed over days 11-13 of incubation 59, after the vessels had matured but not yet stiffened (making microbubble injection less feasible), and the motions of the embryo were still minimal. It should be noted that typically more than 50% of the embryos were lost prior to experimentation due to contamination, yolk breakage, or other environmental circumstances 60.

### Experimental system

An apparatus was configured to enable concurrent intravital high-speed imaging and passive cavitation detection of ultrasound-stimulated microbubbles in CAM vasculature (***Figure 1***).

*Optical apparatus.* For intravital imaging, a microscope was connected to a high-speed camera (APX-RS Photron Fastcam, Tucson, AZ, USA) and controlled with the Photron FastCAM Viewer Software (PFV). Brightfield illumination of the targeted region was achieved using a high-intensity mercury lamp (X-Cite 120Q, 120W; Lumen Dynamics, ON, Canada) coupled to an optical fiber and a lens tube containing a 15 mm focal distance plano-convex lens sandwiched between two 8 mm condenser lenses (LA1540 and ACL12708U, Thorlabs, Newton, NJ, USA). This arrangement enabled the focusing of the light beam at a 15 mm offset without significant loss of intensity, providing sufficient light at the CAM surface for continuous high-speed imaging of single vessels at 10k frames/s with a shutter speed of 2 µs. The fast shutter speed enabled the acquisition of clear images of the bubbles by limiting the temporal averaging.

High-speed images were then obtained at two different optical scales with long working distance water immersion objective lenses. The first set of images was acquired at a smaller field-of-view (FOV) (0.66 mm x 0.66 mm) on a single vessel scale at 10k frames/s with a shutter speed of 2 µs using a 40x objective (LUMPLFLN40XW, NA = 0.8, WD = 3.3 mm, FN = 26.5 mm; Olympus, Tokyo, Japan). This image sequence revealed microbubble dynamics and interactions with the vessel wall on a micro-spatiotemporal scale. The second set of images was acquired on a larger FOV (2.65 mm x 2.65 mm) at 3k frames/s with a shutter speed of 1 µs using a 10x objective lens (UMPLFLN10XW, NA = 0.3, WD = 3.5 mm, FN = 26.5 mm; Olympus, Tokyo, Japan). Under these imaging conditions, several (~50) vessels of different calibers were evident, revealing the statistical nature of the cavitation-induced effects on a vascular network scale with a compromise in the spatiotemporal resolution. This dataset was further exploited to quantify the relative frequency of events.

An additional camera (Pentax KP, Ricoh Imaging Company, Tokyo, Japan) was mounted for co-aligned imaging of the CAM at a lower frame rate of 60 frames/s for up to 5 min. This was achieved by the addition of a broadband non-polarizing cube beam-splitter (Model 13-419, Edmund Optics, Barrington, NJ, USA) modified to be inserted inside the camera tube (U-TRU side camera port, Olympus, Tokyo, Japan). Utilizing this apparatus, the light beam was divided into two paths, with the majority of light going into the Photron high-speed camera and a small amount into the low frame rate Pentax camera. This enabled high-speed, short timescale acquisitions (100s of ms) of microbubble activation to be accompanied by lower frame rate, but longer timescale (2 min) observations of the same FOV, revealing more sustained effects.

*Ultrasound apparatus.* For concurrent sonication and intravital imaging, an in-house PZT-4 ring transducer (~1 mm focal depth, -6 dB beam width of ~1 mm) 61 was attached to the bottom of a glass coverslip (15 mm diameter, 150 µm thickness) with cyanoacrylate glue and then was placed in a custom objective-ring coupler (***Figure S1A***) consisting of an aluminum housing mounted on a 1” lens tube 62. This objective-ring coupler enables ultrasound propagation co-axial to the objective lens without obstructing the light path and simultaneously maintains the alignment of optical and acoustic foci. This setup is further distinctly different from other acoustic high-speed microscopy studies where the transducer is typically positioned orthogonal or at an angle to the optical axis, considerably limiting ultrasound transmission to shorter pulse lengths of lower therapeutic relevance to avoid reflections from the objective.

For sonication, the ring transducer was air-backed, with a 70 µL droplet of deionized and degassed water in the inner area for water-coupling with the objective lens. The ring transducer was then matched to 50 Ω impedance, 0° phase load with a driving frequency of ~1 MHz in thickness mode, and pressure calibrated using a fiber-optic hydrophone (10 μm active element, FOHSv2, Precision Acoustics Ltd., Dorchester, UK) with the objective lens in place in the custom objective-ring coupler to mimic experimental conditions. The ring transducer created a focal region at an average depth of 1 mm from the surface with a -6 dB lateral beam width of about 1 mm (***Figure S1B***). A single 5 ms sinusoidal pulse at 1.04 MHz was generated with peak negative pressures of 0.5-3.5 MPa (corresponding to mechanical indices MI of 0.49-3.43) in 0.5 MPa increments using an arbitrary waveform generator (AFG3102, Tektronix, Beaverton, OR, USA), attenuated by 20 dB (HAT-20+, DC 2 GHz, Mini-Circuits, Brooklyn, NY, USA), and amplified by a 50 dB radiofrequency power amplifier (2100L, 0.1-12 MHz, 100 W; Electronics Innovation Inc., Rochester, NY, USA) before being transmitted through the ring transducer.

Acoustic emissions were passively detected using a spherically focused broadband receive transducer (C302-SU, 1” diameter, 1.63” focal length, center frequency of 1 MHz, -3dB bandwidth of 0.16-1.84 MHz Olympus, Tokyo, Japan) situated at a 50º angle relative to the axis of ultrasound propagation and beneath the custom dish. The orientation was chosen via geometric calculations based on the receiver focal spot size, and the dimensions of the water tank with all of the components in place. This receiver was chosen due to its relatively broad bandwidth and ability to capture both sub- and ultra-harmonics (above and below the driving frequency). Received signals were attenuated by 10 dB (HAT-10+, DC 2 GHz, Mini-Circuits, Brooklyn, NY, USA) and then amplified with a 35 dB preamplifier (AU-1583, 0.3-200 MHz; MITEQ Inc., Hauppauge, NY, USA) prior to digitization at 125 MHz with a 14-bit PC-based oscilloscope (PicoScope 5000D, PicoTechnology Ltd., Cambridge, UK).

*Spatial and temporal co-alignment.* The spatial co-alignment of the optical FOV and acoustic focus of the ring transducer in the z-axis was accomplished with the custom objective-ring coupler by adjusting the lens tube positioning. For alignment in the x-y focal plane, an Opticell cell culture chamber (Nunc, Thermo Fisher Scientific, Rochester, NY, USA) filled with a diluted solution of Definity microbubbles (Lantheus Medical Imaging, North Billerica, MA, USA) was utilized. Upon sonication, the microbubbles were pushed to the circumference of a circle associated with the main lobe of the acoustic field. The relative position of the transducer was then manually fixed to align the center of the optical FOV with the center of the sonicated circle using the translational mounts (LM1XY, Thorlabs, Newton, NJ, USA). Spatial co-alignment of the transmit and receive transducers was then achieved by maximizing the pulse-echo responses from a phantom in the x-y plane holding two perpendicular 27G needles in x and y directions, followed by a more precise alignment with a 500 µm diameter glass bead.

Temporal synchronization between the optical high-speed imaging, ultrasound transmit, and cavitation detection was achieved with a second arbitrary waveform generator (AFG3022B, Tektronix, Beaverton, OR, USA) and a pulse delay generator (575 Series Pulse Generator, Berkeley Nucleonics Corp., San Rafael, CA, USA). On the single vessel scale (40x objective, 10k frames/s), the camera and digitizer were triggered 25 ms prior to sonication and continued recording for a total of 75 ms (25 ms pre-sonication + 5 ms pulse length sonication + 45 ms post-sonication). To capture more sustained effects, the camera and digitizer were triggered again 1 s later to record another 75 ms. On the vascular network scale (10x objective, 3k frames/s), 500 ms was recorded in total (80 ms pre-sonication + 5 ms pulse length sonication + 415 ms post-sonication). Cavitation signals were also recorded accordingly for a total duration of 50 ms (30 ms pre-sonication + 5 ms pulse length sonication + 15 ms post-sonication). These timings were chosen based on the memory limitations of the optical system to accommodate capturing both pre- and post-sonication events.

### Experimental procedure

Between days 11-13 of incubation, a healthy embryo with an intact yolk sac, clear albumin, and a CAM substantially grown over the albumin was chosen for imaging. Prior to the experiments, lipid-shelled Definity microbubbles (Lantheus Medical Imaging, North Billerica, MA, USA) were activated at room temperature via mechanical agitation with a VialMix activation device (Lantheus Medical Imaging) for 45 s and set to passively cool to room temperature. The vial was then gently resuspended and allowed to decant for 2 min for passive size separation 63. A volume of 50 µL of Definity was extracted using an 18G needle and diluted in 0.95 mL of room temperature saline. Finally, 100 µL of the diluted microbubble solution (5 µL of Definity microbubbles) was injected under the microscope into one of the veins of the CAM using a custom glass needle with a 60 µm sharp tip. Estimating a blood volume in a duck embryo on day 12 to be about 2 mL 64, the injected volume of Definity will result in a concentration of about 2.5x10^7^ microbubbles/mL. This is equivalent to 10x the FDA-approved clinical diagnostic dose for humans, and is within the range of other microbubble studies using the CAM model (injections of 3.5-10 µL of microbubbles at earlier stages of the embryo) 48, 65, 66, other animal models in the context of drug delivery and antivascular ultrasound (10^6^-10^10^ microbubbles/mL) 23, 37, 67, as well as emerging clinical trials of antivascular ultrasound 68, 69.

Immediately after the injection, an appropriate region of interest was chosen on the CAM surface (~4 cm^2^) and covered with a thin mylar film (76 µm) to stabilize the surface and enable water coupling with the objective lens. The prepared embryo was immediately transferred to the experimental setup for imaging. The dish was fixed on a stage controlled by a 3-axis motorized stage with <1 µm precision (Thorlabs, Newton, NJ, USA) inside a tank filled with deionized, degassed water at 34°C. 1 mL droplet of degassed and deionized water was placed on top of the mylar film fixed on the CAM surface. The dish was then translated upwards in the z-axis for water coupling with the coverslip of the transducer (held in the objective-ring coupler, and similarly water-coupled to the objective lens), and was then finely adjusted into the optical (and therefore also acoustic) focus. At this point, the whole optical and acoustical system was triggered by the BNC delay generator. Upon completion, the dish was translated in the x-y plane to a fresh region of the CAM, and the procedure was repeated. The time between microbubble injection and experimental acquisitions was recorded: The first acquisition was captured at 5 min post-injection to allow sufficient time for homogeneous microbubble distribution in the CAM 48, 66. To account for the circulation time of microbubbles, imaging for each embryo was limited to 20 min post-injection. In addition, to minimize the effects of potential temporal and spatial inhomogeneities, only a specific region of the embryo's vascular system was imaged for all of the FOVs (the edge of the CAM tissue).

Optical data were acquired on both single vessel and vascular network scales with the associated acoustic signal recorded. The high-speed images obtained on the single vessel scale revealed details of vascular events with micron-scale spatial resolution. It should be noted that the radial oscillations of microbubbles and other rapid effects such as fragmentation, collapse, and microjets cannot be observed due to limitations in frame rate and the frame exposure time (1-2 µs). The small FOV at this scale also limits observations to a small region of a single vessel resulting in the inability to capture events on the edge or outside of the FOV. Therefore, to address the limitations of this spatial scale, another set of data was acquired at a vascular network scale, allowing for the statistical nature of events (and downstream effects) to be captured more effectively, albeit with a compromise in spatiotemporal resolution. It should be noted that the CAM model is considered to possess a generally homogenous structure 70 and that the acquired FOVs were not found to significantly differ from one another in terms of vessel spatial and size distribution. A total of 59 embryos were imaged: 47 at the single vessel scale (619 FOVs), and 12 at the vascular network scale (109 FOVs). The total number of acquisitions (FOVs) at each pressure along with the subset that captured vascular events (microbubble activation, microbubble extravasation, wall deformation, RBC leakage, blood flow directionality changes, clot formation) are summarized in ***Figure S1***.

### Data analysis

*Optical data.* The acquired high-speed images were analyzed using the Photron FastCAM Analyzer 4 software (PFV4). On the single vessel scale, vessel diameters were manually measured pre-sonication, over the first ms of sonication, and after 1 s from the sonication onset (post-sonication). These measurements were performed locally at the site of visible microbubble activation, and perpendicular to the local tangent of the vessel wall boundary. In cases of vessel wall damage at this site, the boundary was found by extrapolating the adjacent observable boundary line. Based on these measurements, the rapid (i.e. during microbubble cavitation) and sustained (i.e. 1 s post-sonication, after the microbubble has left the vessel) vessel deformations were calculated. Rapid deformation was defined as the largest absolute value of the vessel diameter changes during sonication, normalized to the initial vessel diameter. Sustained deformation was defined as the maximum diameter change at 1 s post-sonication, normalized to the initial vessel diameter. Deformations could be symmetric constrictions and dilations of a vessel segment, or they could be asymmetric invaginations or expansions of the vessel wall that were localized to the vicinity of microbubble activation. A large majority of cases were local asymmetric wall deformations, but the data for all deformation types was grouped for the purposes of analysis. It should be noted that this analysis was non-viable in cases where images lacked enough clarity for boundary detection or where the FOV became visually occluded at later times due to hemorrhage.

A qualitative description of the blood velocity field within selected vessels was obtained using a particle image velocimetry (PIV) approach. Here, the RBCs were considered to be particles of interest for tracking purposes. The video frames were first preprocessed using ImageJ 71-73, to become more amenable to PIV. Pre-processing consisted of suppressing vertical stripes via removal of the real axis in the image frequency domain, gaussian filtering, contrast histogram equalization, application of a maximum filter of radius 2, and lastly thresholding via the Niblack binarization method. The open-source MATLAB package PIVlab was then used to obtain blood flow velocity field estimates. A three-pass approach was utilized, with successive interrogation windows (64×64, 32×32, and 16×16 px) having 50% overlap. Note that frame-skipping was employed for sequences corresponding to slow flow in order to minimize sub-pixel interpolation errors. It should be noted that frames containing vessel motion, namely those coinciding with microbubble activation, were excluded.

On the vascular network scale, initial vessel diameter, vessel type (arteriole vs. venule), and proximity of events to bifurcations were measured. Vascular events were recorded for each vessel and categorized as microbubble activation (upon sudden appearance of visibly large bubbles), microbubble extravasation (microbubble extravasation vs. microbubble activation and wall interaction event without extravasation), RBC leakage (recovered or sustained RBC leakage vs. microbubble activation/extravasation without RBC leakage), and flow reversals (transient vs. sustained flow reversal). It is of note that in some cases, microbubble activation was visualized, but subsequent microbubble extravasation events were classified as 'unclear' due to an optical focus loss or temporary vascular deformation beyond the FOV. Proximity to bifurcations was classified as events occurring within 50 μm of a branching point, where this distance refers to the length between the centroid of the event and the crossing of the centerlines of the vessels. It should be noted that events were manually counted; to reduce bias, clear classifications for each event were outlined, and two authors counted and sorted all events.

For quantification, pressure groups were combined as follows: Single vessel data (vessel wall deformation events) was assessed at low pressures (1 MPa), intermediate pressures (2, 2.5 MPa), and high pressures (3, 3.5 MPa), as outlined in ***Figure S1***. Vascular network data (events of microbubble activation, extravasation, RBC leakage, and flow reversal) was assessed at low pressures (≤1 MPa indicating 0.5, 1 MPa), intermediate pressures (2 MPa), and high pressures (3 MPa). The pressures employed were primarily based on prior studies within our group where the range from 1-3 MPa (for 1 MHz, 5 ms pulses) captured the onset of antivascular effects and the transition to pronounced antivascular shutdown 24, 35, 40, 41, along with the observed level of severity of bioeffects found here. Data was normalized by the total number of vessels in each caliber bin and pressure group unless indicated otherwise.

*Acoustic data.* Acoustic data was post-processed in MATLAB (MathWorks). Cavitation was assessed on the vascular network scale as the larger optical FOV aided in correlating the optical (2.65 mm x 2.65 mm) and acoustic (2.5 mm beam width at -6 dB) data. Received signals were digitally filtered with a 5^th^-order bandpass Butterworth filter (0.3 MHz high-pass and 10 MHz low-pass) and were further processed using two approaches. In the first method, intended to evaluate overall cavitation within the pulse, the signal was multiplied by a 500 µs rectangular window starting at the sonication onset (offset = 0 µs). In the second approach, intended to assess the temporal evolution of the cavitation signal during the sonication pulse, 200 µs sliding Hanning windows starting at 0 µs and moving by 100 µs increments (50% overlap) were utilized. In both cases, a noise comparison was performed by applying the same window length to the received signal prior to the arrival of the ultrasound pulse. Windowed signals were then zero-padded to a frequency resolution of 200 Hz per division prior to computing the Fourier transform and subsequent power spectral density (PSD). This analysis was performed for sonications with microbubbles present in each experimental embryo, yielding the power spectra for microbubble cavitation signals (PSD_signal_) as well as before ultrasound sonication with microbubbles present (PSD_noise_). Both signals were normalized by the mean power of noise in a bandwidth of f_0_-3f_0_ where f_0_ is the fundamental frequency. It should be noted that normalization was done to noise levels rather than the baseline due to a lack of proper baseline acquisition before microbubble injection and prior to sonicating each FOV.

For quantification of cavitation peaks, PSD_signal_ and PSD_noise_ were integrated over 10 kHz frequency bandwidths centered at the peak point values of subharmonic 1/2f_0_, ultraharmonic 3/2f_0_, as well as 1/3-order sub- (1/3f_0_+2/3f_0_) and ultra- (4/3f_0_+5/3f_0_) harmonics. Broadband noise levels were also calculated by taking the average of the integrated power spectra over a 40 kHz frequency band centered at 3.42f_0_ and 3.58f_0_ away from the peaks but within the bandwidth of the receiver, similar to previous studies of high-speed microbubble behavior 57 and of antivascular ultrasound *in vivo* 24 under similar exposure conditions as the present study. The relative peaks were quantified as the ratio of mean power of the peaks to the surrounding broadband levels calculated in bandwidths of (0.45-0.47)f_0_+(0.53-0.55)f_0_ for 1/2f_0_ subharmonic, (1.45-1.47)f_0_+(1.53-1.55)f_0_ for 3/2f_0_ ultraharmonic, (0.28-0.3)f_0_+(0.36-0.38)f_0_ for 1/3f_0_ subharmonic, (0.61-0.63)f_0_+(0.69-0.71)f_0_ for 2/3f_0_ subharmonic, (1.28-1.3)f_0_+(1.36-1.38)f_0_ for 4/3f_0_ ultraharmonic, and (1.61-1.63)f_0_+(1.69-1.71)f_0_ for 5/3f_0_ ultraharmonic. Statistical significance was evaluated with a right-tail one-sided t-test performed on the power of each peak (assuming equal variances) across different pressure groups. To compare the level of each peak to the noise level, a similar test was performed between the signal and noise peaks calculated from PSD_signal_ and PSD_noise_, respectively.

Finally, the correlation between the optical and acoustic data was evaluated by plotting the percentage of occurrence of RBC leakage per FOV (number of vessels with signs of RBC leakage is divided by the total number of sonicated vessels in the specific FOV for 3 vessel sizes of 5-20 µm, 20-50 µm, and ≥50 µm) against the integrated power of broadband noise and other sub- and ultraharmonic peaks.

## Results

### Optical observations

**Figure 2:**
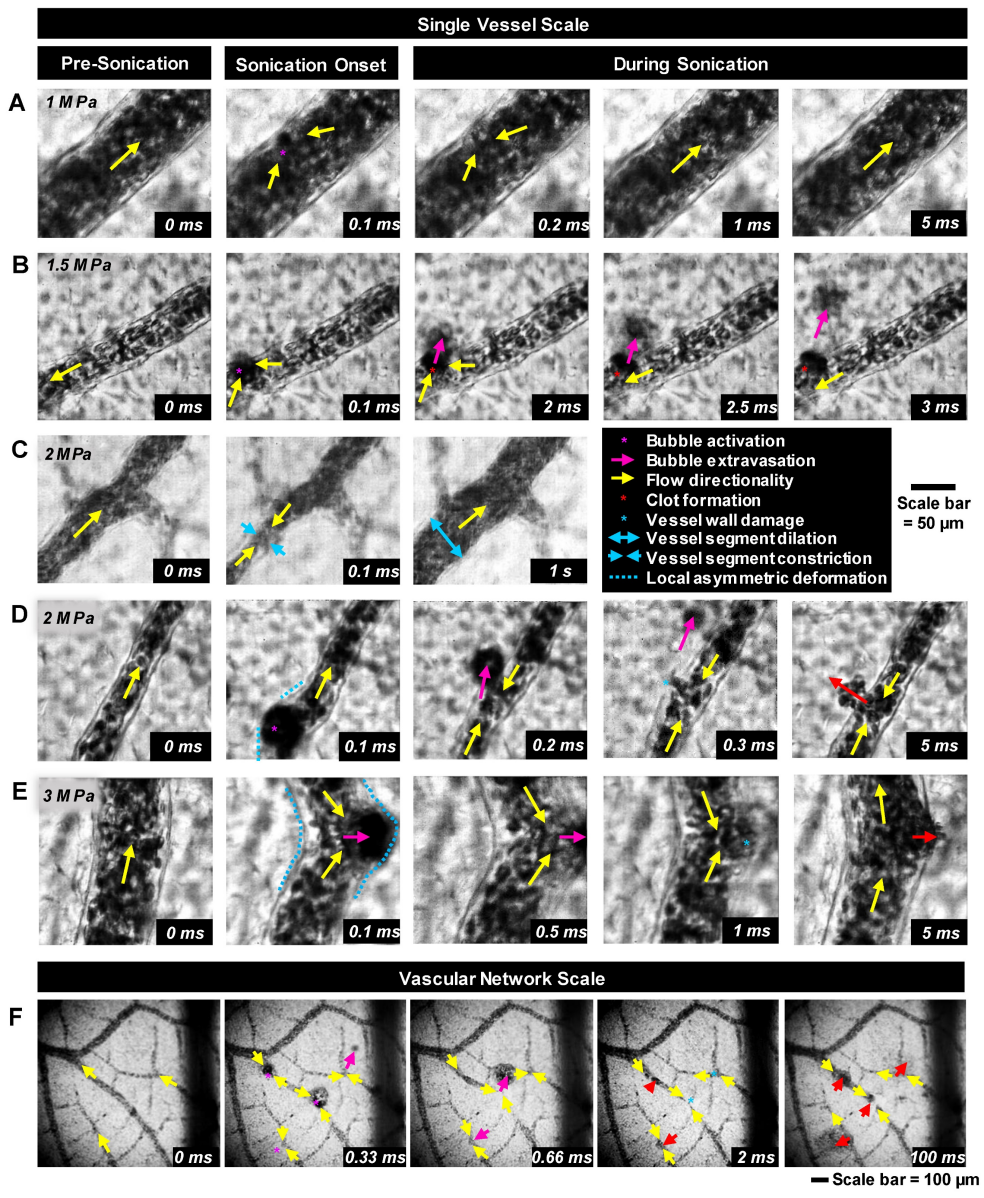
** Bubble-vessel interactions. (A-E)** Series of images captured on the single-vessel scale for a range of pressures in different vessel calibers. **(A)** 1 MPa: Microbubble activation and collapse, followed by brief flow reversal with minimal interaction with the boundary of a 94 µm diameter vessel. **(B)** 1.5 MPa: Microbubble activation, extravasation, and brief flow reversal in a 44 µm diameter vessel. A clot forms at the site of disruption, and flow directionality recovers. **(C)** 2 MPa: Brief microbubble activation in a 46 µm diameter vessel followed by flow reversal, vessel segment constriction, and then dilation and flow directionality recovery. **(D)** 2 MPa: Microbubble activation in a 41 µm diameter vessel, extravasation with a sustained wall disruption, flow reversal, and hemorrhage. **(E)** 3 MPa: Microbubble activation with large local asymmetric vessel wall deformation, brief flow reversal, and leakage in an 83 µm diameter vessel. **(F)** Example images captured on the vascular network scale sonicated at 3.5 MPa showing multiple points of microbubble activation and extravasation followed by vessel damage and RBC leakage. The complete set of videos associated with these examples can be found in Supplementary Videos S1-S6.

High-speed images of ultrasound-stimulated (1.04 MHz, 5 ms pulse length, peak negative pressures of 0.5-3.5 MPa) Definity^TM^ microbubbles were captured in CAM vessels (5-300 µm diameter) on the single vessel (∼0.2 mm^2^, 10k frames/s, 2 µs shutter speed starting at the beginning of each frame, 40x objective lens; ***Figure 2A-E***) and vascular network (∼5 mm^2^, 3k frames/s, 1 µs shutter speed, 10x objective lens; ***Figure 2F***) scales. The single vessel scale illustrates the details of microbubble-vessel interactions with a higher spatial resolution, while data acquired on the vascular network scale elucidates the relative frequency of these events. The frame rates employed in this study do not temporally resolve microbubble oscillations, however, they do enable insight into microbubble-microvessel interactions over a longer timescale (5 ms) relevant to typically employed FUS pulses.

Illustrative examples of single vessel image sequences are shown in ***Figure 2A-E*** (with associated videos in the *Supplementary Information*). Before sonication (first column of ***Figure 2***: 0 ms), a vessel is apparent with a clear boundary and RBCs flowing inside of it. Upon sonication onset (second column of ***Figure 2***: 0.1 ms), a stimulated microbubble can become optically apparent relative to surrounding RBCs (termed microbubble activation), appearing suddenly as a large circle with a dark rim. It is important to note that primarily due to the ultrasound exposure conditions employed here (long pulse length, high pressure), the activated microbubble could expand to a significant degree (e.g. ***Figure 2E***). Once a microbubble is activated, it interacts with the vessel inducing wall deformations, followed by a cascade of vascular events that may include microbubble extravasation, RBC leakage, and blood flow alterations.

**Figure 3:**
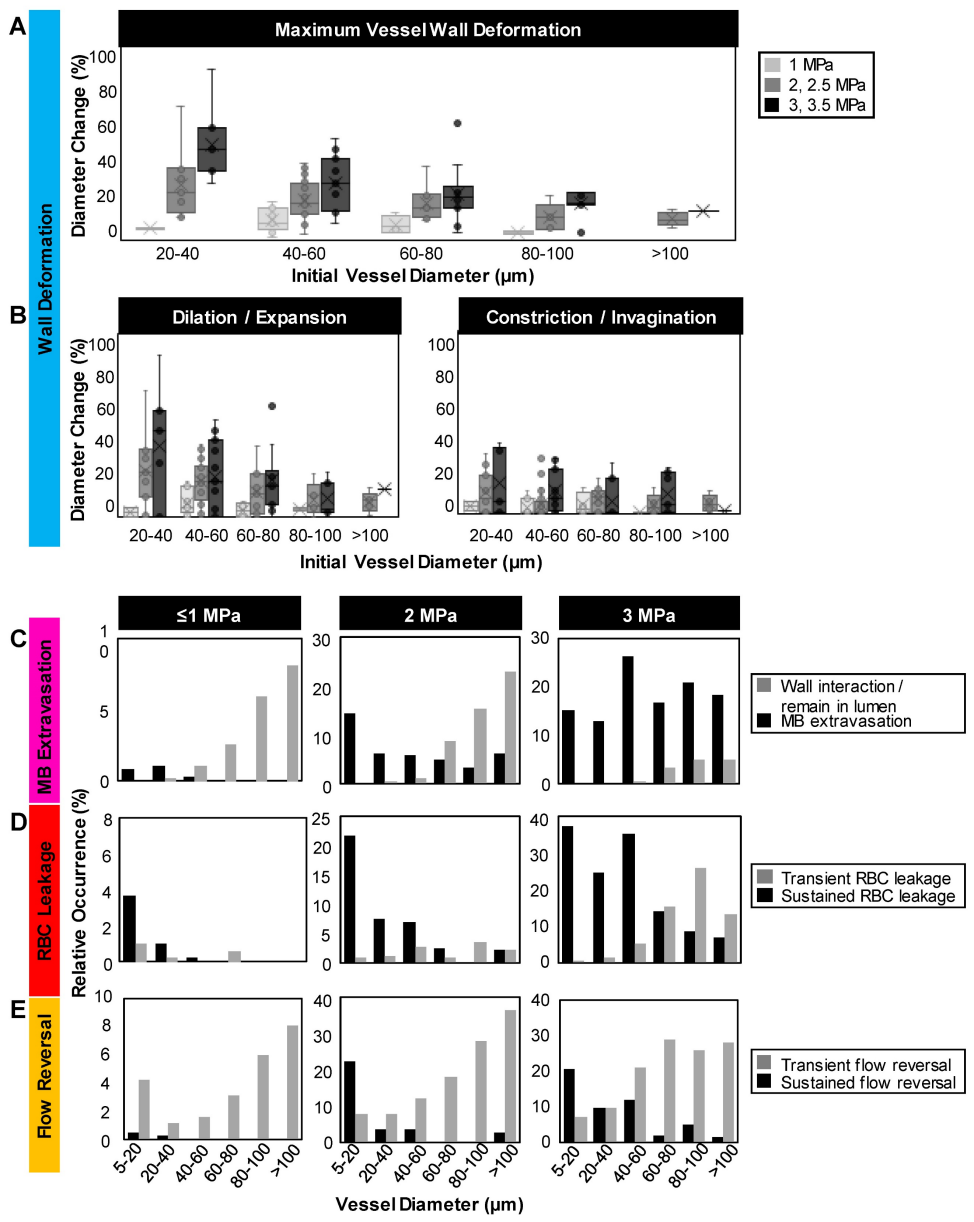
** Quantification of vascular events. (A)** Maximum relative deformation, **(B)** dilation/expansion (left) and constriction/invagination (right) of the vessel wall during the first 1 ms of sonication as a function of pressure and initial vessel diameter. Data quantified from acquisitions at the single vessel scale. **(C)** Histograms of occurrence of microbubble extravasation (wall interaction but remaining in the lumen, or extravasation), **(D)** RBC leakage (transient or sustained), and **(E)** flow directionality reversals (transient or sustained) as a function of pressure and vessel diameter, relative to the total number of (binned) vessels visualized at each pressure. Data quantified from acquisitions at the vascular network scale.

**Figure 4:**
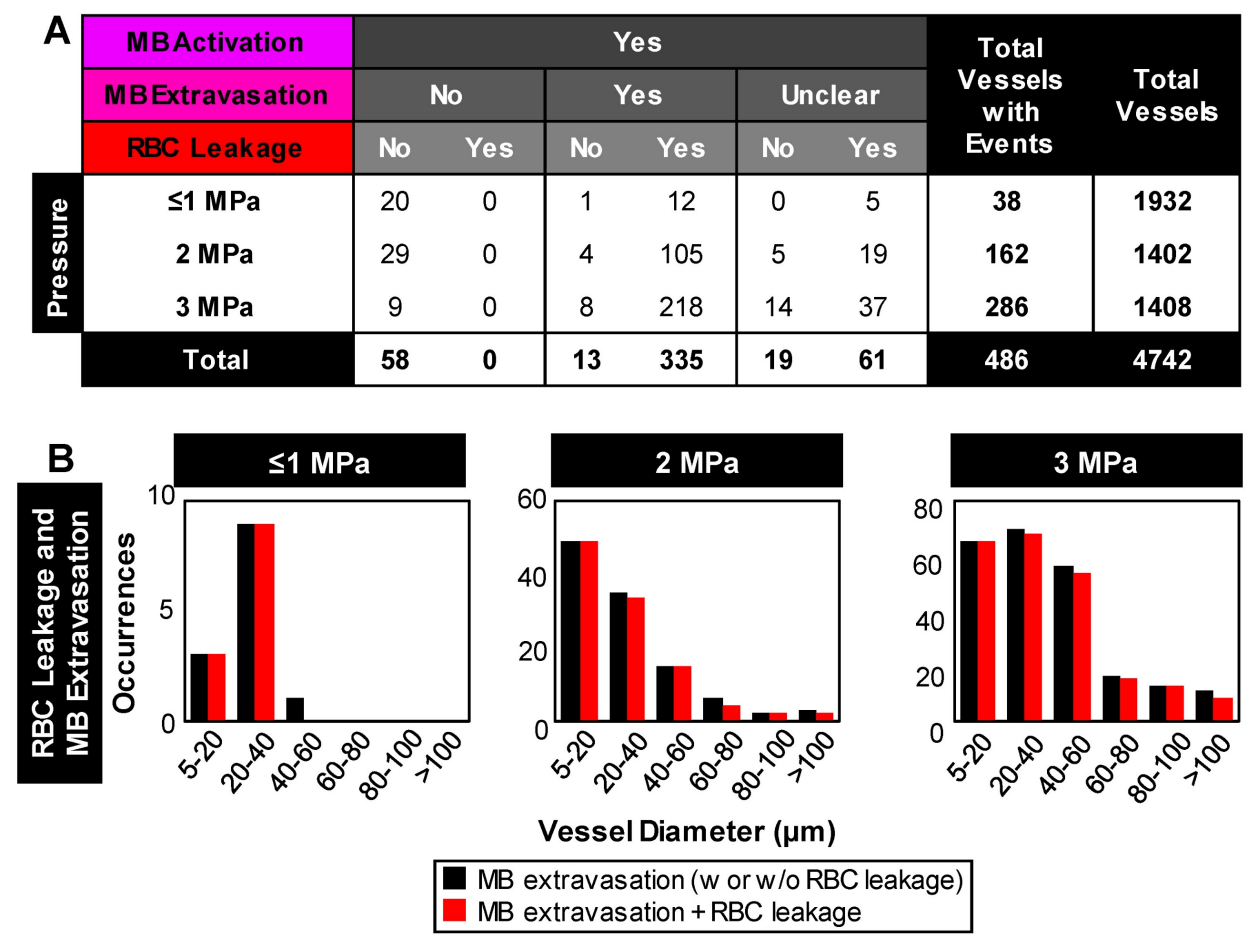
** Relationship between microbubble extravasation and RBC leakage** following microbubble activation. **(A)** Table of event incidences (microbubble extravasation and RBC leakage) following microbubble activation as a function of pressure. Note that cases of microbubble extravasation that were labeled as 'unclear' did not directly capture the event due to an optical focus loss or vascular deformation beyond the FOV. **(B)** Histograms of the relative counts of vessels with RBC leakage in cases of microbubble extravasation as a function of pressure and vessel diameter. Data quantified from acquisitions at the vascular network scale.

For quantification purposes, all visible vessels in the vascular network acquisitions were counted and their diameters were measured, and the subset of vessels exhibiting events were recorded (***Figure 3****, **Figure 4**, **Figure S2***). It is of note that due to the frame rate (temporal resolution) and optical limitations, there were cases with evidence of one vascular event (e.g. RBC leakage) while the details of prior events (e.g. microbubble extravasation) were missed. Overall, microbubble activation was observed in 10% (486/4742) of the vessels and its incidence increased as a function of pressure, from <2% at 1 MPa (38/1932 vessels) to 20% at 3 MPa (286/1408 vessels; ***Figure S3***). It should be noted that variations in the total vessel count at each pressure are due to differences in the number of acquisitions (FOVs) at each pressure, and the distribution of vessels within each FOV (see ***Figure S2***). Further descriptions of subsequent vascular events are given below.

*Vascular deformation.* Microbubble activation events inside the lumen could persist for several frames (~few hundred µs) during which time the microbubble may translate inside the vessel towards the wall. If the microbubble comes into close proximity with the wall, it may interact with it and induce cascading events that can cause vascular deformations that could include constrictions and dilations of the vessel segment or more localized asymmetric invaginations or expansions of the vessel wall (indicated by blue markings in ***Figure 2***).

To quantify these vascular deformations, for vessels with apparent microbubble activity, local maximum changes in vessel diameter were measured during the first 1 ms of sonication. These rapid (within 1 ms of sonication) vascular deformations were quantified as a function of pressure and vessel caliber (***Figure 3A-B***). In general, larger deformations were observed at higher pressures and in smaller vessels, with dilations/expansions occurring to a greater degree and likelihood than constrictions/invaginations. It should also be noted that asymmetrically localized wall deformations comprised a large majority of the cases. At lower pressures (1 MPa), vessel diameters changed by <10% in 70% of vessels, whereas at higher pressures (3, 3.5 MPa), deformation magnitudes increased by >30% relative to the initial vessel diameter in 33% of vessels. Assessing by vessel size and deformation type (dilation/expansion vs. constriction/invagination), it was found that vessels of 20-40 µm in diameter exhibited <5% change at 1 MPa, increasing to (on average) 38% dilation/expansion or 16% constriction/invagination at 3 MPa; vessels of <100 µm in diameter experienced no dilation/expansion or constriction/invagination at 1 MPa, and up to 17% dilation/expansion or 11% constriction/invagination at 3 MPa on average.

*Microbubble extravasation.* After activation, the microbubble may remain inside the microvessel lumen or interact with the vessel wall (e.g. inducing deformations) in a manner that it achieves extravasation or leakage (observed as a bubble interacting with the wall followed by its appearance outside and adjacent to the vessel), typically in proximity to the point of activation. For all cases of microbubble activity at 1 MPa, the bubble remained inside the lumen in 53% (20/38) of cases and extravasated from the vessel in 34% (13/38), with its fate being unclear for the remainder of the vessels (5/38); see ***Figure 3C****,** Figure 4***. At this low pressure, extravasation typically occurred in smaller microvessels, with the largest having a diameter of 56 μm. At 3 MPa, extravasation was more frequent, with only 3% (9/286) of activated bubbles remaining inside the vessel and 79% extravasating (226/286). At this higher pressure, extravasation was observed to occur in larger vessels of up to 180 μm in diameter. Notably, when microbubble extravasation occurred, it had an 88% likelihood of transpiring within the first 500 μs of sonication and >94% prior to 1 ms (***Figure S4***). In rare cases (as in ***Figure 2B***), oscillation against the boundary persisted for a few milliseconds prior to extravasation. Microbubble fate following extravasation could not always be captured, due to limitations of the FOV boundaries or the optical focal plane. Generally following extravasation, the microbubble could be seen traveling outside of the vessel beyond the FOV bounds; or it could become less optically distinct (becoming a circular shadow rather than a circle with a dark rim), which could be due to dissolution or movement out-of-plane.

*RBC leakage.* Following microbubble extravasation, the vessel wall could be compromised, resulting in either a transient (~ms, during sonication) or sustained (~s to mins following sonication) leakage of RBCs. As was observed with microbubble extravasation, RBC leakage incidence rose with increasing pressure and decreasing vessel diameter (***Figure 3D***). At 1 MPa, there was RBC leakage in 45% (17/38) of vessels with apparent microbubble activation events, with said vessels having a mean diameter of 20 μm. At 2 MPa, 76% (124/162) of affected vessels (with a prior microbubble activation event) yielded RBC leakage and larger vessels were involved (beginning to affect vessels of up to 70 μm in diameter, with the mean affected vessel being 26 μm). At higher pressures of 3 MPa, RBC leakage was evident in 89% of cases (255/286), involving still larger vessels of >70 μm with a higher propensity, with an overall mean affected vessel diameter of 34 μm. Intriguingly, a correlation was observed between RBC leakage and microbubble extravasation (***Figure 4***). Among the 58 instances where the bubble did not extravasate, no RBC leakage was detected. Conversely, out of 348 cases exhibiting microbubble extravasation, a subset of 335 incidents were followed by RBC leakage (***Figure 4A***). Importantly, this trend of highly consistent (>90%) RBC leakage following microbubble extravasation was maintained across all pressures and vessel calibers (***Figure 4B***).

**Figure 5:**
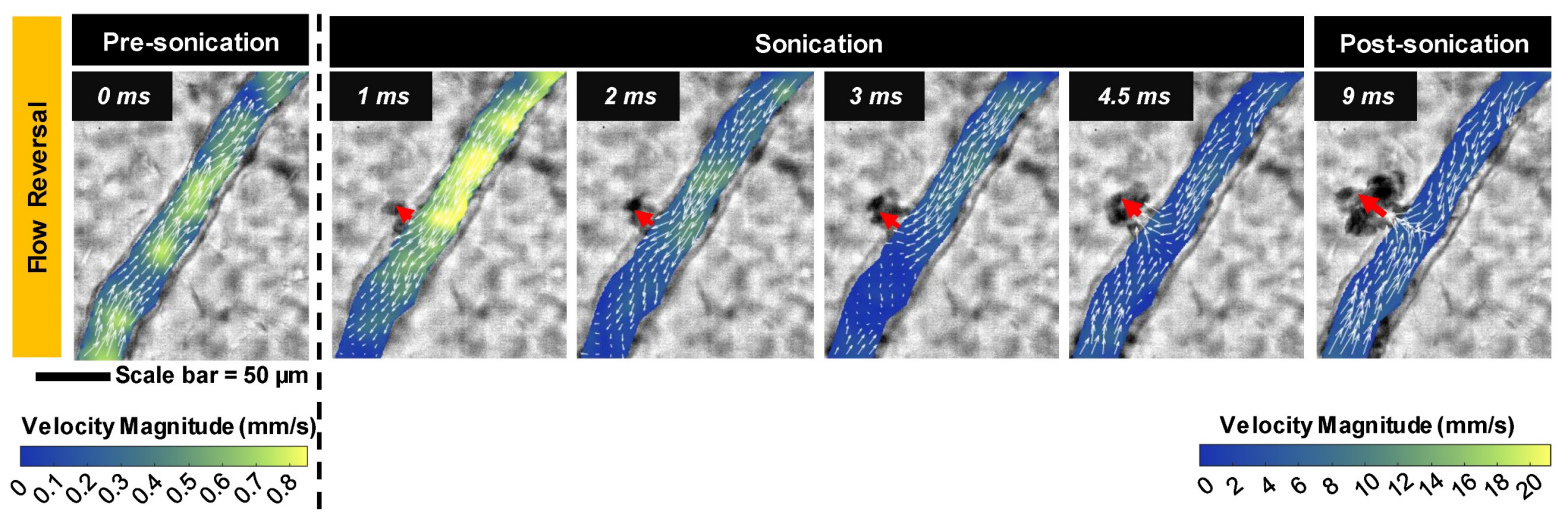
** Blood flow velocity field.** Quantified flow before and after microbubble sonication for an example vessel (same as the vessel in Figure 2D) showing the reversal of flow directionality towards the point of microbubble activation and RBC leakage (indicated by red arrow). Note the change in velocity scale, with a substantial transient surge reaching a peak velocity of 26.8 mm/s at 1 ms after sonication onset. Data was acquired at the single vessel scale.

*Blood flow alterations.* In all cases of microbubble activation, blood flow directionality is immediately altered with RBCs being locally drawn towards the point of microbubble activation/extravasation at an increased speed *(**Figure 5**).* Flow reversal events therefore occurred with a similar propensity to microbubble activation events, increasing with pressure and decreasing with vessel caliber; though with higher overall incidence as flow reversals could additionally be observed in up- and down-stream vessels in the network. Similar to RBC leakage, flow directionality reversals could be classified as transient (~ms) or sustained (~s to min), with sustained reversals always being accompanied by sustained RBC leakage. At 1 MPa, flow directionality reversals were typically transient, with 92% (35/38) exhibiting recovered flow directionality. At 3 MPa, only 56% (160/286) of flow reversals recovered transiently.

Interestingly, it was observed that proximity to bifurcations played a significant role in the incidence of the above vascular events, with >38% of such events (microbubble activation, microbubble extravasation, RBC leakage) occurring within 100 µm of a branching point (***Figure S5A***). This observation was conserved across both arterioles and venules, with there being less than a 10% difference in the rate of event occurrence between them *(**Figure S5B**).*

### Longer timescale effects

The observations made on a longer timescale provided insight into the sustained effects of microbubble cavitation. The cavitation-induced deformations were not limited to short timescales when the bubble was present inside the vessel and they could be sustained long after the bubble has left on the minute timescale (***Figure S6***). Similar to rapid deformations (occurring within 1 ms of sonication; ***Figure 3A-B***), sustained deformations on a second timescale (defined as the global relative diameter change from rest 1 s after sonication onset) show a strong dependence on pressure and vessel size. Remarkably, at 1 s after sonication onset, all the vessels were dilated; however, on a minute timescale, both constriction and/or dilation were observed and accompanied by visible changes in flow rate. Examples can be found in ***Figure S7***, with accompanying supplementary videos. Additionally, in a few cases following microbubble activation, the apparent formation of clots (darker static regions consistent with the aggregation of blood cells sustained over a long period of time) was captured (***Figure S8***). This occasionally led to the cessation of flow in the vessel that would then either recover or persist for the remaining imaging duration.

### Acoustic observations

**Figure 6:**
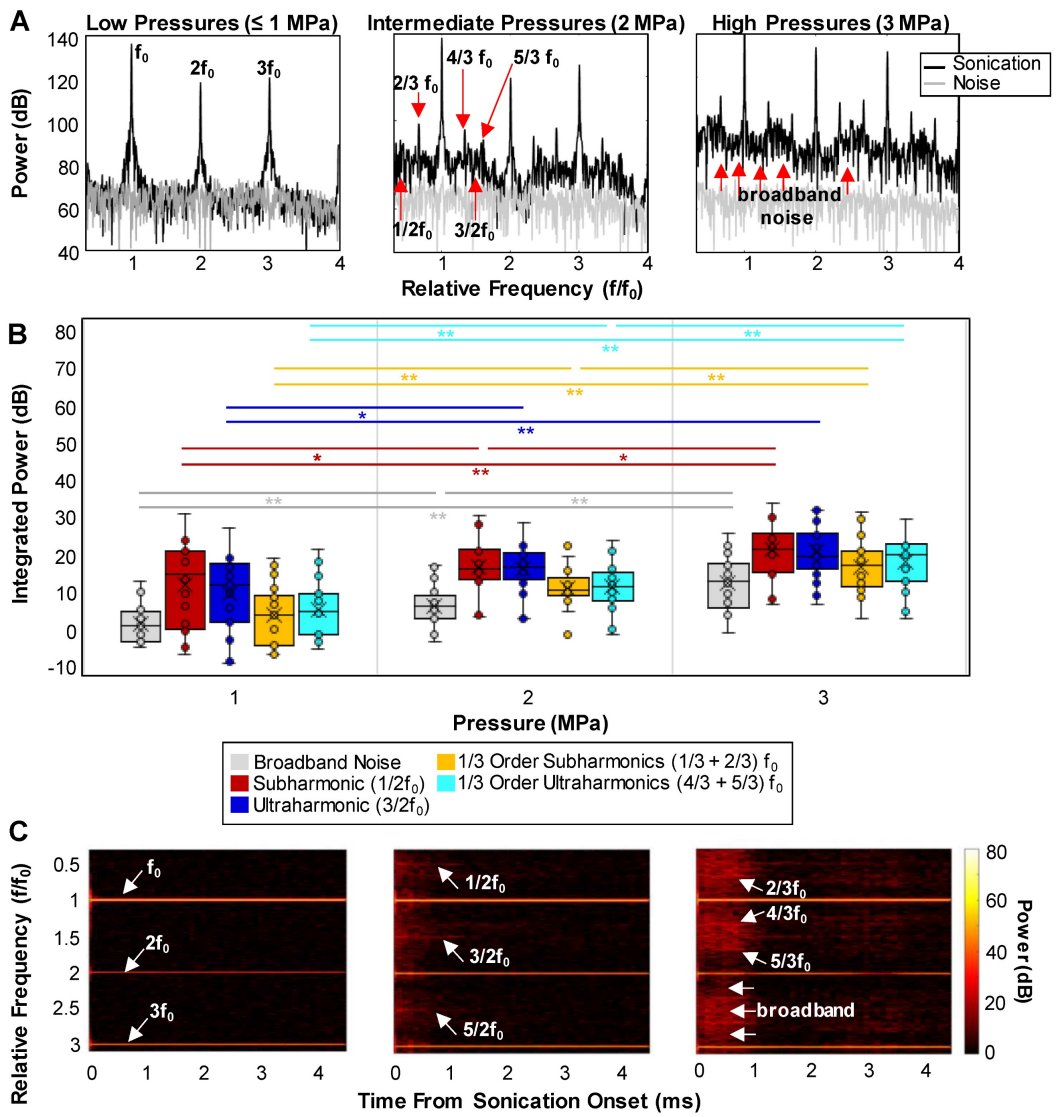
** Cavitation signal. (A)** Examples of detected cavitation spectra at different pressures showing the emergence of sub- and ultra-harmonics along with an increase in the broadband noise level at higher pressures. **(B)** Quantification of the peaks: box and whisker plots showing the integrated power of the broadband noise, 1/3 and 1/2 order sub- and ultra-harmonic peaks as a function of pressure. * = p < 0.05, ** = p < 0.01. **(C)** Examples of the time evolution of the cavitation spectra (spectrograms) at different pressures displaying the persistence of the IC and sub- and ultra-harmonic peaks through sonication time. All cavitation data was quantified from acquisitions at the vascular network scale.

A series of representative cavitation spectra are illustrated in ***Figure 6A*** for different pressures. Typically, at lower transmission pressures (≤1 MPa), peaks at the fundamental frequency (f_0_) and harmonics became evident in the spectra. In the intermediate (2 MPa) and high (3 MPa) pressure groups, broadband noise levels (indicative of inertial cavitation or IC) increased and distinct 1/2 order sub- and ultra-harmonic peaks appeared, along with the interesting emergence of their 1/3 and 2/3 peak counterparts. The quantified cavitation spectra in ***Figure 6B*** show that the increases in the power of IC, 1/2 order subharmonic (1/2f_0_), 1/3+2/3 subharmonic, and 4/3+5/3 ultraharmonic bands were statistically significant between all pressures. In particular, the powers of the second harmonic and 1/3 order ultraharmonic (3/2f_0_) were only significant from 1 to 2 MPa. Quantified cavitation spectra as a function of pressure for the sub- and ultra-harmonic peaks and their third-order counterparts relative to the surrounding IC can be found in ***Figure S9***. A further interesting aspect of the cavitation signal is its evolution as a function of time during the sonication pulse (***Figure 6C***). Collectively, at the very beginning of the pulse (0.05-0.1 ms), high levels of broadband noise with sharp sub- and ultra-harmonic peaks were observed, followed by a quick drop to the noise level in 0.5 ms after sonication onset, with only the fundamental and harmonics persisting. This indicates that a shorter sonication duration may be sufficient to yield microbubble extravasation and RBC leakage events.

The relationship between the optical bioeffects and the acoustic characteristics was also assessed by plotting the occurrence of RBC leakage per FOV as a function of the integrated power of IC and sub- and ultraharmonics (***Figure S10***) for different vessel size groups (5-20 µm, 20-50 µm, ≥50 µm). While a clear linear or threshold-like pattern is not evident in these plots, there is, however, a general increase in the likelihood of RBC leakage as the IC power increases. At lower IC powers (of amplitudes of <200), many vessels remain intact with no RBC leakage present (points falling on the x-axis). As IC power is increased, more vessels become affected and begin to show signs of RBC leakage, particularly in the case of larger vessels.

## Discussion

Here, optical high-speed microscale observations of ultrasound-stimulated microbubbles within microvessels with simultaneous cavitation monitoring were made *in vivo*. A range of acute vascular events were captured during and immediately following sonication, most notably the intact extravasation of microbubbles along with rapid changes in flow directionality and possible subsequent vessel wall disruption and RBC leakage. Further, the variance of the microbubble-induced events was examined as a function of pressure and vessel caliber, exhibiting a substantial increase in event occurrence at higher pressures and in smaller vessels. More sustained effects were also captured on a longer timescale (~mins), showing sustained vessel wall deformations with significant changes in flow rate, but rare occurrences of clot formation and flow cessation. Synchronously detected cavitation spectra illustrated emerging broadband noise and distinct sub- and ultra-harmonic peaks at higher pressures with accompanying third-order peaks.

### Optical observations

A central finding was the first direct *in vivo* optical observation of bubble extravasation during an exposure pulse. The presence of gas-containing structures in the extravascular space is particularly exciting, suggesting that bioeffects can arise from both intra- and extravascular cavitation, extending the potential of microbubble-mediated therapies beyond the vasculature. It is further possible that downstream immune responses triggered by such exposure schemes that are apparent in immunocompetent but not immunodeficient mice 41 might be due to extravascular cavitation. It is thus hypothesized that microbubbles oscillating in the perivascular space can interact directly with tumor cells, and that extravascular cavitation may thereby be a mechanism for immunogenic cell death. Extravascular cavitation may also have implications for drug delivery, in terms of actively promoting drug transport or modifying the mechanical properties of the extravascular space in a manner that impacts subsequent passive transport. The results suggest that efforts should be directed toward understanding the potential role of extravasated microbubbles in therapeutic effects that are observed in preclinical and clinical studies that employ exposure conditions that result in RBC extravasation. It is also of interest to consider the development of exposure conditions that have the objective of enhancing the bioeffects of extravasated bubbles. Previous studies have provided indirect evidence of bubble extravasation; where the extravasated shell material was detected using histology 74 or acoustic signatures of extravascular cavitation were captured 75. This has generally been accomplished in the context of lower pressure (drug delivery) regimes, with nanobubbles hypothesized to be extravasating. However, these approaches were incapable of directly visualizing intact bubbles outside of vessels. In a more direct approach in an *ex vivo* rat mesentery model under high-speed microscopy with short, high pressure pulses (2 cycles, 4-7 MPa), examples were reported of bubbles transitioning through microvessel walls, leaving post-insonation remnants in the extravascular space 53. It was concluded that a degree of wall damage (termed rupture) was necessary for this to occur, supported by observations of endothelial cell detachment and injected dye extravasation. While further reports with this model did not observe extravasated bubbles, considerable evidence indicated that wall damage was associated with both bubble jetting behavior and wall deformations induced by stimulated intravascular microbubbles 51, 53. This is also supported by *ex vivo* observations in the rat cecum model, which reported wall deformations with short exposures at lower pressures (100 µs pulse lengths, 0.8 MPa) 50.

The observations of wall deformations in the present study are therefore consistent with previous reports and may be implicated in inducing a level of damage that enables the extravasation of microbubbles. It should be noted, however, that while the microscopy approach taken here permitted the observation of millisecond timescales, the temporal resolution (frame rate and shutter speed) was insufficient to capture details of the deformation process and possible jetting behavior. A striking difference to the previous *ex vivo* reports - in which only a single study reported examples of extravasation 53 - was the observation of widespread microbubble extravasation events. This may be due to a number of factors, a primary one being the pulse length: millisecond scale pulses were used in the present study, which are typical for microbubble-mediated FUS exposures. Specifically, it is notable that 60% of microbubble extravasation events occurred between 0.2-0.3 ms (36.2% occurring from 0.4-5 ms), and only 3.8% within 0.1 ms of sonication onset (***Figure S4***). This suggests that a sufficient degree of wall damage must accrue over time due to intravascular microbubble behavior to then enable extravasation and that this is facilitated by the use of longer pulses (at the pressure levels employed). A second factor is the potential impact of the presence of RBCs, which were absent in the previous *ex vivo* studies. The impact of RBCs on bubble behavior and wall interactions in microvessels has to date received little attention, though it has been shown that the microvascular wall pressures exerted by bubble oscillations are impacted by the presence of RBCs, most notably in capillaries.

The present results also demonstrate pressure-dependent increases in extravasation incidence in preferentially smaller vessels, consistent with observations of deformation levels (***Figure 3A****, **Figure S6***). Prior studies under other conditions and models further support the present findings, where a preferential impact was captured in smaller microvessels with greater potential for vascular damage at higher pressures 43, 50, 53, 76, 77. In the present work at the lowest pressures (≤1 MPa), microbubble extravasation occurred preferentially within smaller vessels (<40 µm), despite higher levels of observed microbubble activity in larger vessels (***Figure S3***). Higher rates of microbubble activation in larger vessels are expected on the basis of higher microbubble concentrations present, along with reduced levels of damping experienced by microbubbles in larger vessels relative to smaller ones 78, 79. The higher pressure threshold for microbubble extravasation from larger microvessels (≥2 MPa) may be attributable at least in part to considerations of wall structure and thickness. Generally, as vessels become larger, the wall becomes thicker and more structurally sophisticated. Whereas capillaries have a simple wall structure consisting of a single layer of endothelial cells typically 1 µm thick, arterioles and venules have multiple layers and become several micrometers thick, from 5 µm thickness in an 18 µm diameter microvessel to 50 µm thick for larger vessels with diameters exceeding 150 µm 80. Thus, one can expect that for a bubble to extravasate from a large vessel, a much higher force is required, making it more challenging for the microbubbles to penetrate through all three layers in larger vessels compared to degrading the single endothelial layer in capillaries. Indeed, the data suggests that higher pressures are required to incur sufficient damage to thicker microvessel walls in order to permit the extravasation of microbubbles.

The behavior of microbubbles and their interaction with microvessel walls as a function of vessel diameter may also be a significant factor influencing the pressure and vessel size dependence of extravasation. Previous work using small diameter (12, 25, and 195 µm) rigid-walled tubes demonstrated that the boundary conditions presented by channel walls progressively reduce the maximum expansion ratio of insonated microbubbles as channel diameters are decreased 81. This work was extended to optically demonstrate that individual microbubble expansion ratios within *ex vivo* microvessels (≤30 µm diameters assessed, with a mean vessel diameter of 18±7 µm) were reduced relative to those occurring in large (200 µm) tubes 50. It was also shown that despite reduced oscillation amplitudes, the small scale of the vessel lumen enabled bubble-wall interactions such that wall deformations could occur. Notably, this work was carried out at 1 MHz with pulse lengths (2 cycles) much shorter than those employed in the present study and previous antivascular studies. In recent work, the pressure-dependent (0.1-3 MPa) behavior of microbubbles within wall-less microchannel (15, 50, 100, and 200 µm diameters assessed) phantoms were examined using longer pulses (0.1 and 5 ms) 57. A prominent observation was that, at sufficiently high pressures (>1 MPa), bubble clusters (or clouds) could form that occupied the lumens of the 15 and 50 µm (but not 100 and 200 µm) microchannels and induced channel wall deformations. The deformations were observed on both sides of the channel wall, suggesting that substantial circumferential strains could be induced by clusters occupying the vessel lumen. Importantly, at sufficiently high pressures these clusters persisted for the duration of the pulse thereby resulting in sustained wall interactions and deformations. Interestingly, the pressure threshold for cluster persistence (and therefore sustained wall interactions) to occur was higher for the 50 µm channel than the 15 µm channel, suggesting that such clusters may be a possible contributing factor to the higher pressure levels required to induce extravasation in larger microvessels. In contrast, it was observed that in 100 and 200 µm channels, microbubbles were directed via primary radiation forces towards the distal wall, where they were present at increased concentration before dissipating rapidly (<0.3 ms). Their reduced persistence, possibly coupled with not occupying a sufficient portion of the lumen as a cluster to exert sufficient circumferential stresses within the wall at the pressure ranges examined, may be a factor in why RBC extravasation (requiring the wall integrity to be compromised) was not as prevalent as in smaller vessels. In addition to wall deformations, it is also notable that other bubble behavior such as micro-jetting and shockwave emissions associated with microbubbles or clusters may be contributing to wall damage in a manner that depends on pressure and vessel diameter. These behaviors and their roles in eliciting vessel wall damage sufficient to enable extravasation, warrant further investigation in future work. Finally, it is also of interest to understand the possible impact of vessel segment diameter changes (e.g. ***Figure 2C***, which can impact damping experienced by the bubbles) during the course of the ultrasound pulse on bubble behavior and wall interactions.

The potential role of primary radiation forces in inducing microbubble extravasation also warrants comment. The penetration of stimulated microbubbles into gels 62 and blood clots 82, or the walls of relatively large channels 83 under the influence of primary radiation forces has been previously reported and occurs predominantly in the ultrasound wave propagation direction. The prevailing direction of radiation forces for the beam configuration in the present study is in a downward direction. If primary radiation forces were the dominant factor driving extravasation (i.e. 'pushing' microbubbles through the walls), it would be expected that they would preferentially exit downwards. Considering the optical configuration employed, it would be expected that the ability to observe microbubble extravasation occurring on the distal side of a vessel would be obscured by the vessel itself, and additionally by considerations of the optical depth of field. The presence of a membrane below the vessels may capture some bubbles that exit downwards and are redirected laterally, but the prevalence of detected bubbles in a lateral direction along with evidence of lateral microbubble extravasation (***Figure 2***) supports that primary radiation forces do not play a dominant role in the extravasation process.

RBC extravasation was also found to be widespread. The histologic assessment of RBC extravasation is frequently conducted in a research setting 45, 37, and it has become a primary indicator of vascular damage. The observation of RBC extravasation is generally attributed to microvascular wall degradation based on the inference that if RBCs are present outside the vessel, there must necessarily be a form of wall degradation to permit their exit. The occurrence of RBC extravasation following FUS microbubble treatments has also been shown previously in a CAM model using a similar frequency (1, 2.25 MHz) and pressure range (0.5-2.3 MPa), albeit with shorter pulses (10 cycles) 48. While the microscopy configuration employed did not enable observations of microbubbles, anecdotal electron microscopy was performed which revealed endothelial cell detachment from the basement membrane 48. It was posited that jetting behaviors of intravascular bubbles were responsible for wall damage that led to RBC extravasations, though these were not directly detected on electron microscopy. A central finding of the present data was the association of RBC extravasation with microbubble extravasation, where microbubble extravasation preceded 96% of cases with RBC leakage. Following microbubble extravasation, RBCs were observed to exit the microvessel at the same discrete location. This indicates that the microbubble extravasation process created a perforation through the wall of sufficient size to permit the subsequent extravasation of RBCs, driven by hydraulic pressure differences between the vessel lumen and the perivascular space. These observations suggest that incidences of perivascular petechiae (e.g. ***Figure S11***, occurring under the same high 3 MPa high pressure, long pulsing scheme in a murine orthotopic breast tumor model with microbubbles) may be caused by microbubble extravasation events, with RBCs following. The observation of both transient (~s) and sustained (~min) RBC extravasations suggest that some perforations may close on shorter time scales (thrombus formation was not frequently observed). To date, the prevailing perspective of FUS-induced microvascular damage is that it is induced by bubbles that are situated within the vessel lumen.

Though a strong association was found between microbubble extravasation and RBC extravasation, it is also worth raising the question if microbubble extravasation is necessary or just a main route for this occurring. While other possible mechanisms for RBC extravasation may exist, the measurement approach must also be considered in interpreting the results. Specifically, in cases where no microbubble extravasation was observed to precede RBC extravasation, it is possible that this may have been due to an inability to capture microbubble extravasation (e.g. in cases of distal side vessel exits).

The details of microbubble behavior and wall interactions during this process warrant further investigation. In the present study, temporal resolution and shutter speed inhibited the ability to assess the details of this process. However, it should also be noted that relative to the case where microvessels are devoid of RBCs, the visualization of microbubbles *in vivo* presents particular challenges. Specifically, it is notable that, under the conditions of this study, the microbubbles appeared to have a thick, dark rim, or to be fully dark, in contrast to prior *in vitro* high-speed microscopy studies with the same optical parameters 57. It may be that the different models (CAM vs. phantom) are resulting in a difference in optics. However, the dark appearance of the bubbles upon activation may also be caused by RBC attraction to the bubbles. Strong attractive forces between oscillating bubbles and cells have been demonstrated in the literature 54, 55 and it should be acknowledged that the presence of RBCs, which are often missing in high-speed microscopy studies of bubble behavior, may further alter the resultant microstreaming pattern. Indeed, all cases of microbubble activation yielded rapid blood flow directionality reversals, with a local pull of RBCs towards the point of microbubble activation and extravasation, as well as a persistent streaming pattern.

Numerical and experimental *in vitro* studies of fluid flow induced by bubble cavitation near a solid boundary have shown an inward pull of flow towards the point of bubble collapse on very short timescales (~few μs) 84-86. Moreover, a handful of *in vivo* studies have reported that the acoustic cavitation of microbubbles can induce changes in blood flow directionality on a longer timescale (~s to min) 24, 87. While the exact mechanisms responsible for the changes in flow direction are not fully understood, we hypothesize that the pressure gradient generated by either microbubble extravasation or oscillation and collapse inside the vessel can locally pull RBCs towards the point of activity. These flow directionality and rate alterations on the millisecond timescale can further result in changes in shear stress on endothelial cells, driving downstream events such as vascular permeability and inflammation. Indeed, microbubble oscillation-driven fluid flow is known to be a crucial underlying mechanism for a range of induced bioeffects. It should be noted, however, that while the present study exhibited frequent blood flow reversals, this effect was observed much less frequently under similar exposure conditions in mice 24. Further, the latter study as well as other studies of high pressure, long pulsing schemes exhibited blood flow shutdown 28, 29, 35, 40, 41, which was not captured in the present work. Presumably this discrepancy can be explained by an important structural difference between the CAM and other animal models, namely the lack of a solid and dense extravascular tissue matrix surrounding CAM vessels which could modify the effective boundary conditions experienced by the microbubbles. This may suggest a role of the extravascular space in facilitating vascular shutdown, perhaps *via* interstitial fluid pressure levels promoting vascular collapse, or possibly material drawn in from the extravascular space into the vascular compartment. While the possible mechanisms and role of the extravascular compartment in microbubble-mediated vascular shutdown remain to be established, the present data supports that this warrants investigation.

It was also found that the location in the vascular network - particularly the proximity to bifurcations - played a significant role. An increased occurrence of vascular events close to a bifurcation has been reported in a handful of *in vivo* studies 24, 48, 87-89 along with a higher probability of microbubble fragmentation close to junctions *in vitro* 81. While the underlying mechanism for this preferential effect is not fully understood, it can be hypothesized that the trapping, slowing, or fragmentation of microbubbles at bifurcations (due to differences in flow rates in downstream branches, particularly in tumor vasculature; see 90, 91) and/or the significant decrease in microbubble oscillation when far from a fluid reservoir (larger vessels in this case) can result in a higher incidence of cavitation-induced vascular events close to branching points.

### Acoustic observations

The findings of this study suggest that similar to the optically observed vascular events, the cavitation signal depends not only on pressure but also on vessel size. Care must be taken in developing control methods for any microbubble-mediated treatments where the cavitation signal is being collected from bulk tissues, and perhaps alternative methods of detecting microbubble-endothelial interactions might be required particularly in the smallest vessels 3, 50. Here we have collected the cavitation signal from an approximately 2D microvascular network which is more closely correlated with microbubble activity in microvessels, compared to receiving signal from a bulk 3D tissue containing a range of vessel calibers.

In general, inertial cavitation (IC) has been linked to petechiae and vascular damage in vessels 15, 24, 37, and has been associated with ischemic necrosis caused by vascular shutdown in brain tissue 92 and in tumors 35, 40. The implications of vascular damage and RBC leakage have resulted in IC being avoided. The present data has demonstrated, however, that IC as well as 1/3 order sub- and ultra-harmonic peaks might offer additional control potential when seeking to either amplify or avoid microbubble extravasation and subsequent biological effects that may ensue, especially when microvessels are the desired targets. The emergence of 1/3 order peaks has been reported in a few other studies in the context of antivascular ultrasound 24, 28, 40, 41, all under therapeutically relevant schemes (>2 MPa high pressure, 5 ms long pulse, ~1 MHz low frequency) with microbubbles. Based on our previous *in vitro* phantom studies 57 under similar exposure conditions, it has been hypothesized that these 1/3 order acoustic signatures arise mainly due to microbubble cavitation and sustained clusters in microvessels (≤50 μm), and do not correlate with larger vessels (≥100 μm) where microbubble clusters are relatively small and dissipate rapidly as they are only held in place by radiation force 57. It is further suggested that shorter pulses of 1 ms may result in similar outcomes as 10 ms pulses, and perhaps such long pulses are not necessary for achieving the desired biological effects nor are they strictly necessary for feedback purposes 10. We finally note that there was a similar time-course between the drop in the spectral peaks and microbubble extravasation (with over 94% extravasating within 1 ms of the start of sonication). Although these are preliminary findings, they warrant further exploration, particularly of the association between microbubble cavitation-induced bioeffects and distinct acoustic signatures.

### Limitations

While the optical transparency of the CAM membrane enabled the high-speed optical imaging of microbubbles *in vivo*, care must be taken in extrapolating the results to other models. As noted in the Discussion, the CAM model notably has a less solid extravascular space than typical models, which may have a two-fold effect. The first concerns the physical aspects, where the bubble dynamics within the vessel and the fate of the extravasated microbubble can be substantially influenced by the viscoelastic properties of the surrounding structure. The second interrelated aspect is the induced biological mechanisms, including continuous blood leakage into the extravascular compartment as well as rare occurrences of clot formations and flow cessations. This is in contrast to previous studies using similar exposure parameters in the context of antivascular ultrasound, where immediate shutdown of blood flow (within 10s of seconds) has been observed frequently in mice 24. It is also worth mentioning that the vessels in the CAM have no terminal vessels, tips, or sprouts, and are always in a closed cycle, as in healthy human vasculature 59. It should further be noted, however, that the development and integrity of the vessels depend heavily on the stage of the CAM 59; though by the time of experimentation in the present study on days 10-12, the vessels have matured. These factors should be considered when extrapolating results to other models and humans. In general, the observations and trends found in this study were in good agreement with previous findings using different vascular models. This suggests that the CAM might offer stable and consistent experimental conditions with high intra- and inter-individual reproducibility, making it a reliable and appropriate preclinical *in vivo* model to study the vascular responses of microbubble-stimulated treatments 93.

It should be noted that the approach utilized here was generally insensitive to vessels below 10 μm in diameter, though microbubble-induced effects might be even greater in smaller capillaries in humans 50. This insensitivity arose from the coupled effects of a limited optical depth of field and the layered nature of the CAM microvascular structure. Capillaries (≤10 μm) are confined to be within a layer just beneath the chorionic epithelium, whereas larger arterioles and venules (>10 μm) are situated deeper in the mesodermal layer 59. The optical focal zone was generally placed at a depth that permitted the assessment of a wide range of microvessel calibers (arterioles and venules), with capillaries lying out of the focus unless deliberately focusing on the superficial capillary bed (***Figure S12***), where the smallest detectable vessel was 5 µm in diameter. However, in general, the smallest detectable vessels were 20 μm and 10 μm on the single vessel and vascular network scales, respectively.

Additionally, the employed methods could not capture all forms of damage (i.e. damage to individual endothelial cells 16, or wall perforations defined as a technical integrity loss resulting in a hole large enough for leakage of dye but not the exit of RBCs 53) or rapid microbubble effects (i.e. radial oscillations, fragmentation, and microjets). Notably, the term 'wall disruption' in this study was defined as an apparent RBC leakage out of the vessel boundary due to disruption of wall integrity, while indications of 'no damage' do not imply that other forms of bioeffects (e.g., opening of tight junctions and/or damage to individual endothelial cells) were not induced.

## Conclusion

A novel apparatus was developed to obtain high-speed microscale observations of ultrasound-stimulated microbubble-vessel interactions with simultaneous cavitation monitoring *in vivo*. A series of microbubble-induced acute vascular events were captured and were found to occur with higher prevalence in smaller vessels as a function of increasing pressure. A central finding was the direct optical evidence of the creation of micron-sized holes in the vessel wall caused by microbubble extravasation. The uncovering of the strong correlation between RBC leakage and microbubble extravasation further provides mechanistic insight into the process of microvessel rupture, which has been widely observed histologically to occur. The cavitation of microbubbles in the extravascular compartment during the course of insonation may have implications for eliciting bioeffects relevant to the delivery of therapeutic agents as well as for immunotherapy. It will therefore be important to understand the possible contribution of extravasated bubbles to the observed therapeutic effects arising from exposure conditions that induce bubble extravasation in preclinical and clinical settings. In preclinical and clinical work that employs lower pressure amplitudes, or cavitation feedback control approaches that limit microbubble oscillation amplitudes, RBC extravasations are generally not observed. This work has particular implications for circumstances where higher pressure exposures may be employed for delivery, or in the setting of antivascular therapy which is now emerging into clinical trials in oncology.

## Supplementary Material

Supplementary figures and video legends.

Supplementary video 1.

Supplementary video 2.

Supplementary video 3.

Supplementary video 4.

Supplementary video 5.

Supplementary video 6.

Supplementary video 7.

Supplementary video 8.

Supplementary video 9.

Supplementary video 10.

Supplementary video 11.

Supplementary video 12.

Supplementary video 13.

Supplementary video 14.

Supplementary video 15.
