# Supplementary material for: In vivo high-speed microscopy of microbubbles in the chorioallantoic membrane model
Source: Theranostics. 2024 Feb 17;14(5):1794–814. doi: 10.7150/thno.91232 (PMC10945333; doi:10.7150/thno.91232)
Supplement: Supplementary file 1 — Supplementary figures and video legends. [file thnov14p1794s1.pdf]

Supplementary Information

In vivo high-speed microscopy of microbubbles in the chorioallantoic membrane model

Rojin Anbarafshan[1,2,], Carly Pellow[2], Kevin Kiezun[2], Hon Leong[1,2], David E. Goertz[1,2]*
[1]Department of Medical Biophysics, University of Toronto, Toronto, M5G 1L7, Canada
[2]Sunnybrook Research Institute, Toronto, M4N 3M5, Canada

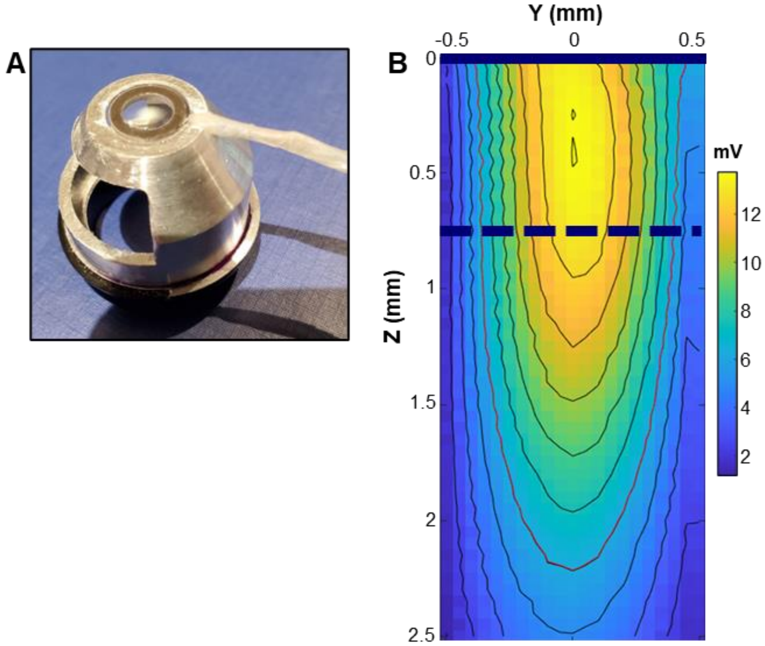

Figure S1. Overview of the sonication apparatus. (A) *The custom objective lens–ring transducer coupler.* ***(B)*** *Pressure field generated by the ring transducer. The thick dashed line indicates the approximate position of the CAM surface.*

Single Vessel Scale

A

Pressure (MPa)	Acquisitions (FOVs)	Affected Vessels
**1**	134	22
**2**	172	51
**2.5**	113	17
**3**	159	34
**3.5**	41	5

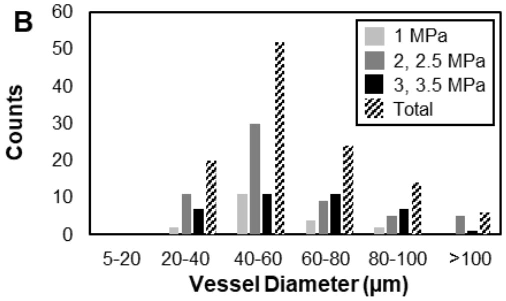

Vascular Network Scale

C

Pressure (MPa)	Acquisitions (FOVs)	Vessels	Affected Vessels
**0.5**	10	508	1
**1**	32	1424	51
**2**	34	1402	204
**3**	33	1408	397

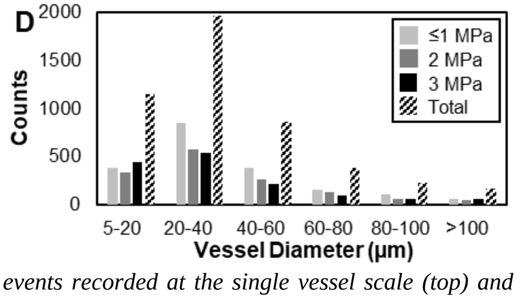

Figure S2. Summary of acquisitions *(FOVs) and events recorded at the single vessel scale (top) and vascular network scale (bottom). Pressure groupings for analysis are indicated in* ***(A)*** *for the single vessel scale, and histograms of affected vessels analyzed in* ***(B)****; and are indicated in* ***(C)*** *for the vascular network scale, with histograms of all visualized vessels (affected and not affected) quantitatively assessed in* ***(D)****.*

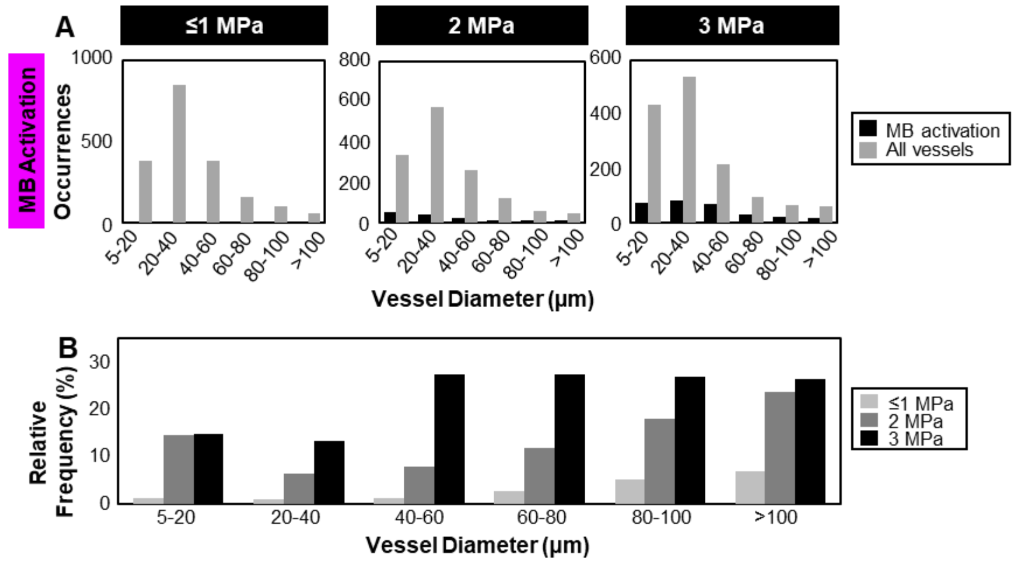

Figure S3. Quantification of microbubble activation. (A) *Histogram of microbubble activation events (black), and total visualized vessels (grey) as a function of vessel diameter at pressures of ≤1 MPa (0.5, 1 MPa), 2 MPa, and 3 MPa.* ***(B)*** *Frequency of microbubble activation events relative to total visualized vessels as a function of vessel diameter and pressure. Data quantified from acquisitions at the vascular network scale.*

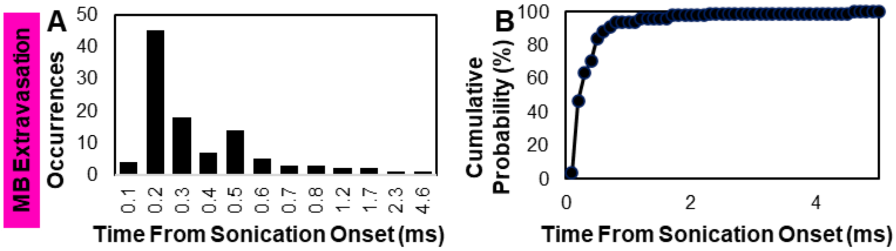

Figure S4. Timeline of microbubble extravasation. (A) *Histogram of the time since sonication onset for microbubble extravasation events (note frames are acquired every 0.1 ms, with a shutter speed of 2 µs).* ***(B)*** *Cumulative probability of microbubble extravasation as a function of time from sonication onset. Data quantified from acquisitions at the single vessel scale.*

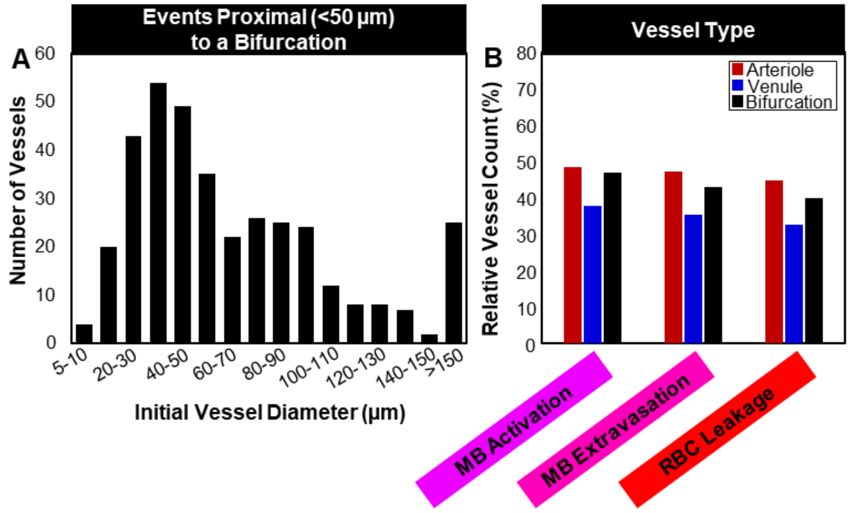

Figure S5. Effect of vessel type and distance to bifurcations on the vascular events. (A) *Histogram of vessels with events occurring proximal (within 50 µm) to a bifurcation.* ***(B)*** *Relative occurrences of vascular events as a function of vessel type (arteriole or venule), and proximity (within 50 µm) to a bifurcation. Events occurred with similar frequency in arterioles and venules, with 40-50% of events occurring close to a bifurcation. Data quantified from acquisitions at the vascular network scale.*

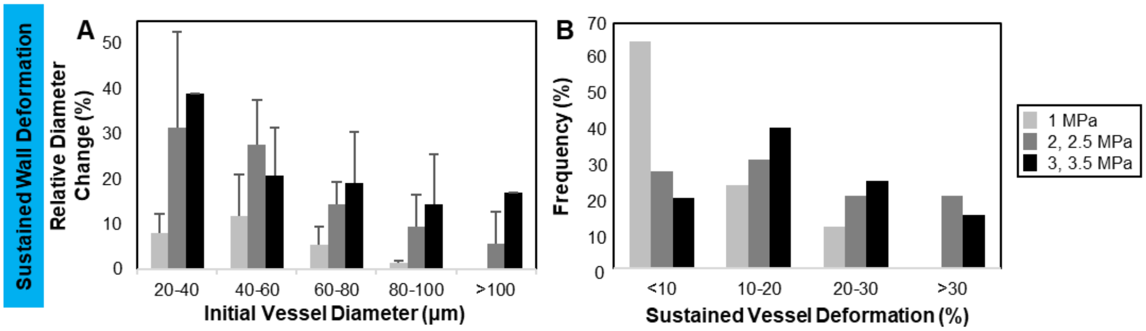

Figure S6. Quantification of sustained (1 s after sonication onset) vascular deformations. (A) *Relative sustained diameter deformation (dilation) as a function of initial vessel diameter and pressure.* ***(B)*** *Frequency of sustained vessel deformation as a function of deformation percentage and pressure. Data quantified from acquisitions at the single vessel scale.*

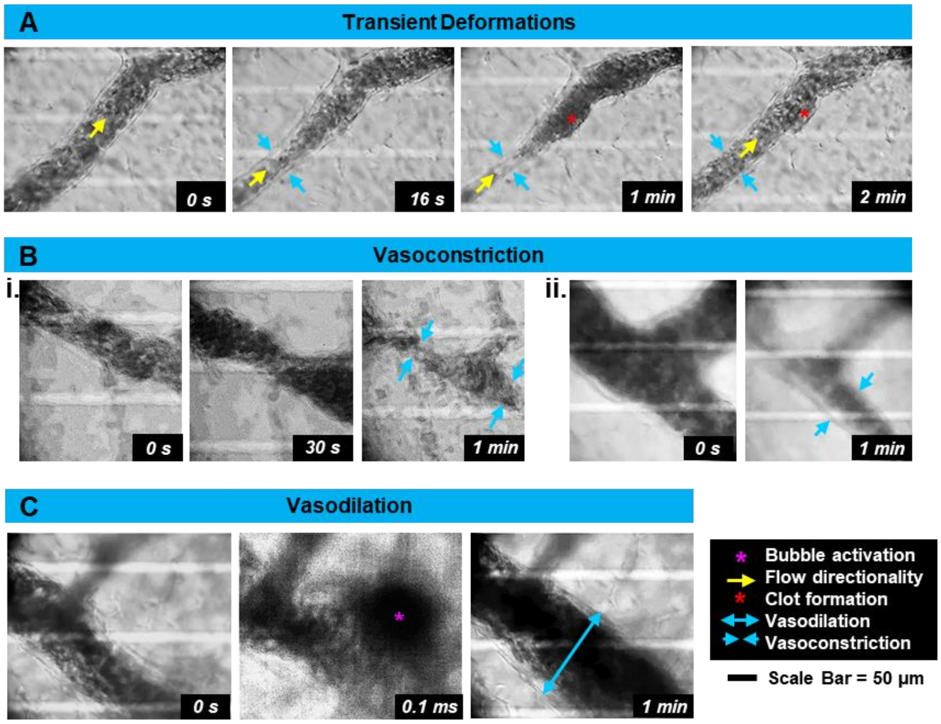

Figure S7. Cavitation-induced vascular deformation on a minute timescale*, visualized at the single vessel scale.* ***(A)*** *Vasoconstriction of vessels due to microbubble activity and induced hemorrhage (i) inside the visualized vessel, resulting in local vasoconstriction, or (ii) downstream, resulting in global vasoconstriction effects (Supplementary Videos S7 and S8).* ***(B)*** *Example vessel with vasodilation with the middle figure showing the microbubble activation event captured by the high-speed camera (Supplementary Video S9).*

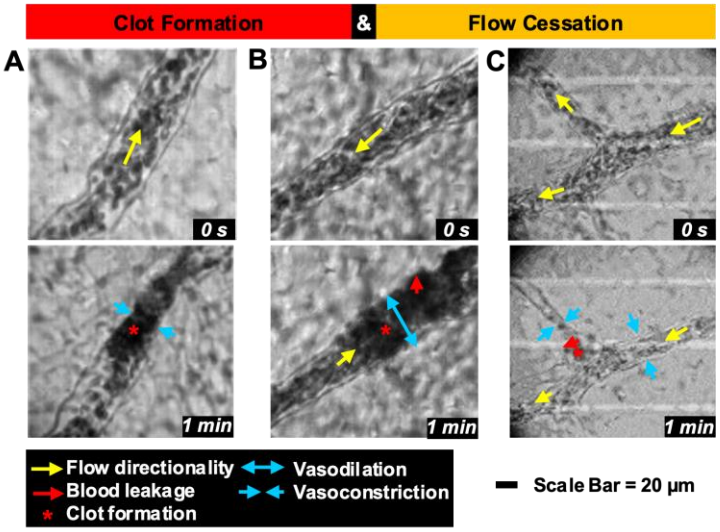

Figure S8. Clot formation and flow changes. *Pre- and post-sonication images captured at the single vessel scale.* ***(A)*** *Clot formation in the vessel with vasoconstriction and significantly decreased flow rate (Supplementary Videos S10 and S11).* ***(B)*** *Formation of a clot with vasodilation and flow decrease (Supplementary Video S12).* ***(C)*** *Complete cessation of blood flow due to downstream events in the marked branch with no blood cells being apparent inside of it (Supplementary Video S13).*

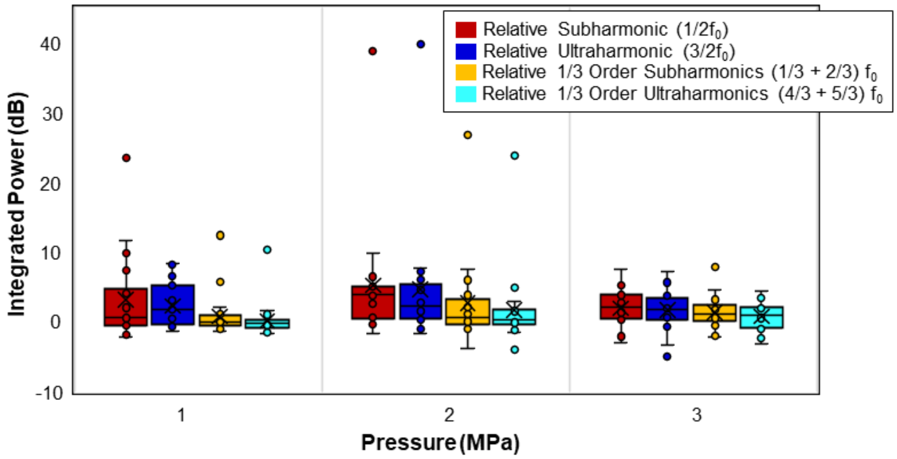

Figure S9. Relative cavitation peaks. *Quantification of the relative peaks calculated as the ratio of the integrated power of subharmonic, ultraharmonic, and 1/3 order sub- and ultra-harmonic peaks to the surrounding broadband noise level at each peak as a function of pressure. All cavitation data was quantified from acquisitions at the vascular network scale.*

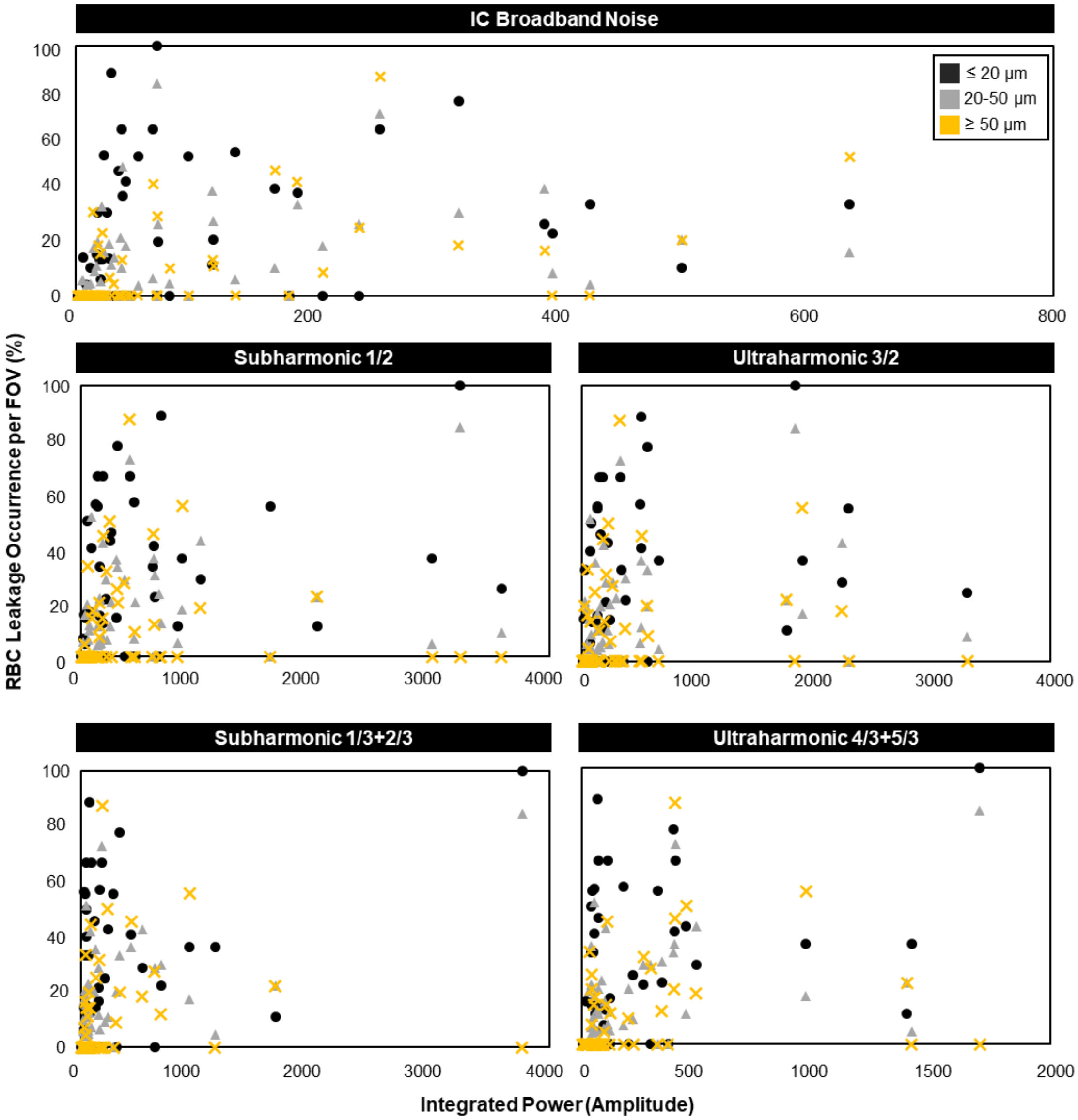

Figure S10. Relationship between bioeffects and acoustic peaks. *The percentage of occurrence of RBC leakage per FOV for different vessel size groups as a function of the power of broadband noise, sub- and ultraharmonic peaks, showing a general increase in the occurrence of bioeffects with increases in the power of cavitation peaks especially for larger vessels. All cavitation data was quantified from acquisitions at the vascular network scale.*

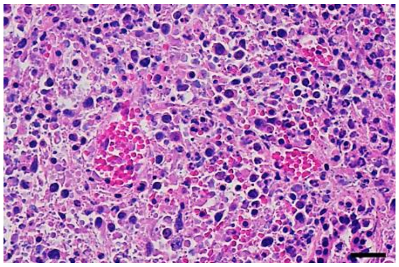

Figure S11. Histology after antivascular ultrasound *(3 MPa, 10 ms pulse length, 5 min duration) in a murine orthotopic breast tumor model, demonstrating perivascular petechiae upon treatment (hematoxylin and eosin staining, scale bar = 50 µm).*

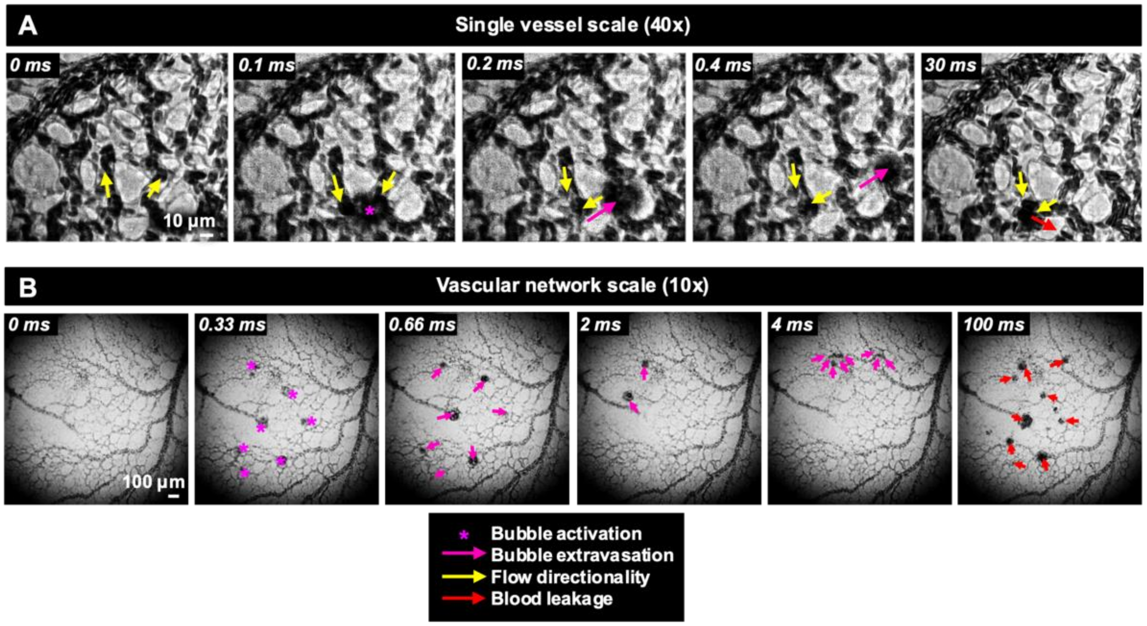

Figure S12. Capillary bed. (A) *Example image of the capillary network captured on the single vessel scale and sonicated at 2 MPa showing the microbubble activation inside one of the capillaries leading to sustained flow reversal and blood leakage (Supplementary Video S14).* ***(B)*** *Example image sequence of the superficial capillary bed under 3 MPa sonication acquired on the vascular network scale illustrating multiple points of microbubble activation and extravasation followed by RBC leakage, indicating sustained local disruption of vascular wall integrity. Some of the extravasated microbubbles continue to oscillate inside the FOV until the end of the pulse (Supplementary Video S15).*

Supplementary Videos: See accompanying .mp4 files for Videos S1-S14

Video S1. *Bubble-vessel interactions in a 94 µm diameter vessel under 1 MPa sonication captured on the single vessel scale, depicting microbubble activation and collapse, followed by a brief flow reversal with minimal interaction with the boundary. Video captured on the single vessel scale (10k fps, 2 µs shutter speed).*

Video S2. *Bubble-vessel interactions in a 44 µm diameter vessel under 1.5 MPa sonication captured on the single vessel scale, depicting microbubble activation, extravasation, and brief flow reversal. A clot forms at the site of disruption, and flow directionality recovers. Video captured on the single vessel scale (10k fps, 2 µs shutter speed).*

Video S3. *Bubble-vessel interactions in a 46 µm diameter vessel under 2 MPa sonication captured on the single vessel scale, depicting brief microbubble activation followed by flow reversal, vasoconstriction, and then vasodilation and flow directionality recovery. Video captured on the single vessel scale (10k fps, 2 µs shutter speed).*

Video S4. *Bubble-vessel interactions in a 41 µm diameter vessel under 2 MPa sonication captured on the single vessel scale, depicting microbubble activation and extravasation with a sustained wall disruption, as well as flow reversal and hemorrhage. Video captured on the single vessel scale (10k fps, 2 µs shutter speed).*

Video S5. *Bubble-vessel interactions in an 83 µm diameter vessel under 3 MPa sonication captured on the single vessel scale, depicting microbubble activation with vessel deformation, brief flow reversal, and leakage. Video captured on the single vessel scale (10k fps, 2 µs shutter speed).*

Video S6. *Bubble-vessel interactions captured on the vascular network scale under 3.5 MPa sonication showing multiple points of microbubble activation and extravasation followed by vessel damage and RBC leakage. Video captured on the vascular network scale (3k fps, 1 µs shutter speed).*

Video S7. *Cavitation-induced vascular deformation on a minute timescale, showing local vasoconstriction of a vessel due to microbubble activity and induced hemorrhage. Video captured at the single vessel scale through the second CCD camera (60 fps).*

Video S8. *Cavitation-induced vascular deformation on a minute timescale, showing global vasoconstriction due to RBC leakage downstream (outside the FOV). Video captured at the single vessel scale through the second CCD camera (60 fps).*

Video S9. *Cavitation-induced vascular deformation on a minute timescale showing sustained vasodilation at the site of microbubble activity. Video captured at the single vessel scale through the second CCD camera (60 fps).*

Video S10, S11. *Clot formation visible on a minute timescale inside the vessel with vasoconstriction and significantly decreased flow rate. Video S10 shows the vessel during the sonication and Video S11 is captured at 1 minute post sonication. Video captured at the single vessel scale through the second CCD camera (60 fps).*

Video S12. *Formation of a clot on a minute timescale accompanied by vasodilation and flow decrease. Video captured at the single vessel scale through the second CCD camera (60 fps).*

Video S13. *Complete cessation of blood flow observed inside the upper left branch due to downstream events with no visible blood cells in the lumen on a minute timescale. Video captured at the single vessel scale through the second CCD camera (60 fps).*

Video S14. *The capillary bed captured on the single vessel scale under 2 MPa sonication showing microbubble activation inside one of the capillaries leading to sustained flow reversal and blood leakage. Video captured on the single vessel scale (10k fps, 2 µs shutter speed).*

Video S15. *The superficial capillary bed captured on the vascular network scale under 3 MPa sonication illustrating multiple points of microbubble activation and extravasation followed by RBC leakage, indicating sustained local disruption of vascular wall integrity. Some of the extravasated microbubbles continue to oscillate inside the FOV until the end of the pulse. Video captured on the vascular network scale (3k fps, 1 µs shutter speed).*
